# Stem cell senescence drives age-attenuated induction of pituitary tumours in mouse models of paediatric craniopharyngioma

**DOI:** 10.1038/s41467-017-01992-5

**Published:** 2017-11-28

**Authors:** Jose Mario Gonzalez-Meljem, Scott Haston, Gabriela Carreno, John R. Apps, Sara Pozzi, Christina Stache, Grace Kaushal, Alex Virasami, Leonidas Panousopoulos, Seyedeh Neda Mousavy-Gharavy, Ana Guerrero, Mamunur Rashid, Nital Jani, Colin R. Goding, Thomas S. Jacques, David J. Adams, Jesus Gil, Cynthia L. Andoniadou, Juan Pedro Martinez-Barbera

**Affiliations:** 1 Developmental Biology and Cancer Programme, Birth Defects Research Centre, UCL Institute of Child Health, London, WC1N 1EH UK; 2 Basic Research Department, Instituto Nacional de Geriatría, Anillo Periférico 2767, Magdalena Contreras, 10200 Mexico City, Mexico; 3 Department of Histopathology, Great Ormond Street Hospital for Children, London, WC1N 3JH UK; 4 Cell Proliferation Group, MRC Clinical Sciences Centre, Imperial College London, Hammersmith Campus, Du Cane Road, London, W12 0NN UK; 5 Experimental Cancer Genetics, Wellcome Trust Sanger Institute, Hinxton, Cambridge, CB10 1SA USA; 6 GOSgene, Genetics and Genomic Medicine, UCL Institute of Child Health, London, WC1N 1EH UK; 7 Ludwig Institute for Cancer Research, Oxford University, Old Road Campus, Headington, Oxford, OX3 7DQ UK; 8 Centre for Craniofacial and Regenerative Biology, King’s College London, Guy’s Hospital, Floor 27 Tower Wing, London, SE1 9RT UK; 9 Department of Internal Medicine III, Technische Universität Dresden, Fetscherstaße 74, 01307 Dresden, Germany

## Abstract

Senescent cells may promote tumour progression through the activation of a senescence-associated secretory phenotype (SASP), whether these cells are capable of initiating tumourigenesis in vivo is not known. Expression of oncogenic β-catenin in Sox2+ young adult pituitary stem cells leads to formation of clusters of stem cells and induction of tumours resembling human adamantinomatous craniopharyngioma (ACP), derived from Sox2− cells in a paracrine manner. Here, we uncover the mechanisms underlying this paracrine tumourigenesis. We show that expression of oncogenic β-catenin in Hesx1+ embryonic precursors also results in stem cell clusters and paracrine tumours. We reveal that human and mouse clusters are analogous and share a common signature of senescence and SASP. Finally, we show that mice with reduced senescence and SASP responses exhibit decreased tumour-inducing potential. Together, we provide evidence that senescence and a stem cell-associated SASP drive cell transformation and tumour initiation in vivo in an age-dependent fashion.

## Abstract (web summary)

Senescent cells can promote tumour progression through the activation of a senescenceassociated secretory phenotype (SASP). Here, the authors show that SASP activation is associated with non-cell autonomous cell transformation and tumour initiation in an in vivo model of adamantinomatous craniopharyngioma.

## Introduction

Cellular senescence defines a state of stable and long-term loss of proliferative capacity, but with retention of normal metabolic activity and viability^1^. The activation of the senescence programme acts as a potent tumour suppression mechanism through the activation of the p53 pathway and expression of cell cycle inhibitors (e.g. p21 (CDKN1A) and p16 (CDKN2A))^2, 3^. The mitogenic stimuli caused by the expression of several oncogenic proteins, including mutant β-catenin, BRAF^V600E^ or KRAS^G12D^, trigger DNA replication stress leading to DNA damage, activation of a DNA damage response (DDR) and the induction of senescence (named oncogene-induced senescence, OIS)^4, 5^. As a result, senescent cells activate a molecular programme characterised by the expression and secretion of a multitude of growth factors, matrix proteases and pro-inflammatory proteins collectively referred to as the senescence-associated secretory phenotype (SASP)^6^. The composition and intensity of the SASP response can be affected by factors such as the senescence-inducing mechanism, cell type and time passed since senescence initiation, suggesting that the SASP is not a singular state^7–10^. The activation of the SASP requires a persistent DDR and is mediated by the NF-κB and C/EBPβ pathways^11^. SASP-associated cytokines, IL-6 and IL-8, reinforce the senescence growth arrest, at least in some senescent cells^12, 13^, which is beneficial in cancer suppression. However, the paracrine activities of senescent cells through SASP activation can also promote tumourigenesis. Prominent or persistent SASP activation has been shown to: (1) disrupt cell–cell adhesion and induce epithelial-to-mesenchymal transition and invasiveness^14, 15^; (2) cause local inflammation^12, 16^; (3) modify tissue architecture^17, 18^; (4) facilitate development of hepatic cancer after carcinogen exposure^19, 20^; (5) stimulate proliferation of nearby pre- and malignant cells both in vitro^21^ and in vivo when co-injected with senescent cells in xenograft mouse models^17, 18, 22^ and (6) favour the emergence of tumour-initiating cells in cell culture models^23–26^. This bulk of evidence demonstrates a pro-tumourigenic role for the SASP, but whether the SASP can induce cell transformation and tumour initiation of non-tumorigenic cells in vivo remain less clear.

We have previously shown that the expression of a degradation-resistant form of β-catenin in Rathke’s pouch, the embryonic primordium of the anterior pituitary gland (*Hesx1*^*Cre/+*^*;Ctnnb1*^*lox(ex3)/+*^ mice)^27^, or in Sox2+ adult pituitary stem cells (*Sox2*^*CreERT2/+*^*; Ctnnb1*^*lox(ex3)/+*^ mice)^28^ leads to the formation of tumours that resemble human adamantinomatous craniopharyngioma (ACP). Interestingly, targeting expression of this mutant β-catenin to cell-lineage progenitors or differentiated cells in the developing pituitary is not tumourigenic, suggesting that the oncogenic effect requires an undifferentiated stem/cell precursor^27^. ACPs are clinically aggressive tumours associated with high morbidity and significant premature mortality^29^. Most human ACPs carry mutations in β-catenin leading to the over-activation of the WNT/β-catenin pathway^30–33^. In agreement with this finding, cells showing nucleo-cytoplasmic accumulation of β-catenin and activation of the WNT pathway are present in mouse and human tumours, commonly grouped in whorl-like structures, named ‘cell clusters’, near the invasive front^29^. These cell clusters are not found in any other type of pituitary tumours^34^, express stem cell markers^27, 35^ and have been proposed to play a critical role in controlling the infiltrative behaviour of surrounding tumour cells^36^.

Although murine clusters derive from mutant Sox2+; S100B+ adult pituitary stem cells expressing oncogenic β-catenin^28^, this population is not the cell-of-origin of the tumours, suggesting a non-cell autonomous role during tumourigenesis. Currently, the molecular and cellular mechanisms underlying the pro-tumorigenic role of this peculiar cell population remain to be discovered. In this study, we demonstrate through molecular and genetics approaches that murine and human clusters are functionally equivalent structures, which show a molecular signature of cellular senescence and a SASP. Our results indicate that tumour induction only occurs in the presence of robust SASP activation, therefore providing evidence for a role of senescence and SASP in tumour initiation in vivo.

## Results

### Pituitary embryonic precursors can induce paracrine tumours

We previously showed that the expression of oncogenic β-catenin in Sox2+ pituitary stem cells in *Sox2*^*CreERT2/+*^*;Ctnnb1*^*lox(ex3)/+*^*;R26*^*YFP/+*^ mice at 4–6 weeks of age results in the formation of pituitary tumours in a non-cell autonomous manner^28^. We sought to investigate whether a similar mechanism may underlie the development of pituitary tumours in *Hesx1*^*Cre/+*^*;Ctnnb1*^*lox(ex3)/+*^*;R26*^*YFP/+*^ mice, another model for human ACP, in which oncogenic β-catenin is expressed in the embryonic pituitary precursors that normally give rise to all the cell lineages of the mature pituitary gland, including Sox2+ stem cells^27^.

**Fig. 1:**
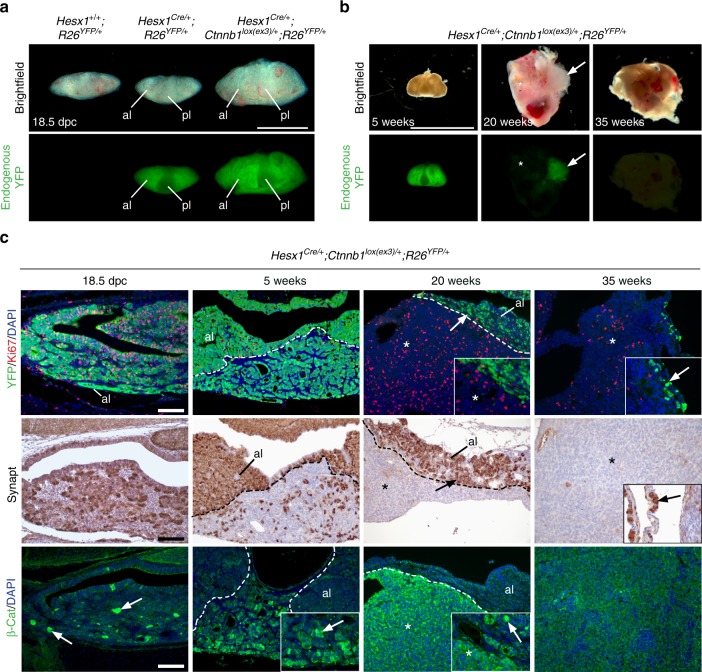
Tumours in the *Hesx1*^*Cre/+*^*;Ctnnb1*^*lox(ex3)/+*^*;R26*^*YFP/+*^ mice form non-cell autonomously after a latency period. **a** Brightfield and epifluorescence images of 18.5 dpc pituitaries. Note that most of the anterior lobe (al), but not the posterior (pl), is fluorescent in both the *Hesx1*^*Cre/+*^*;R26*^*YFP/+*^ control and *Hesx1*^*Cre/+*^*;Ctnnb1*^*lox(ex3)/+*^*;R26*^*YFP/+*^ pituitaries. The mutant pre-tumoural pituitary is enlarged relative to controls. Scale bar: 1 mm. **b** Brightfield and epifluorescence pictures of a pre-tumoural pituitary and tumours in *Hesx1*^*Cre/+*^*;Ctnnb1*^*lox(ex3)/+*^*;R26*^*YFP/+*^ mutant mice at 5, 20 and 35 weeks of age. Most of the tumour cells are not fluorescent and the YFP+ population (white arrow) is displaced to the periphery of the growing tumour mass (white asterisks). Scale bar: 5 mm. **c** Histological characterisation of pre-tumoural pituitaries and developing tumours from 18.5 dpc to 35 weeks postnatally. At 18.5 dpc, most of the embryonic pituitary is comprised of YFP+ cells (revealed with anti-GFP immunostaining), but there are islands of YFP− cells (DAPI+ areas). Abundant Ki67+ cells are distributed throughout the pituitary at this stage. At 5 weeks of age, the pre-tumoural pituitary remains mostly quiescent with very few Ki67+ cells. Note the presence of a dorsal region resembling the normal pituitary tissue of the anterior lobe (al) and a ventral part where YFP− and YFP+ cells are intermingled. The boundary between dorsal and ventral regions is indicated with a dashed line. At 20 weeks, the YFP− population contains abundant Ki67+ cells and has expanded dramatically (white asterisks) displacing the mostly quiescent YFP+ cells to the periphery. Around 35 weeks, mutant mice reach a humane end-point and tumours are mostly YFP− (asterisk). Note that some YFP+ cells are observed in the periphery (white arrow). Immunohistochemistry against synaptophysin shows that whilst the anterior lobe cells (al) are positive, tumours do not express this endocrine marker (black asterisk). Immunostaining against β-catenin showing the presence of cell groups (clusters, white arrows) with nucleo-cytoplasmic accumulation of β-catenin mostly at 18.5 dpc and 5 weeks of age. Subsequently, most of the tumour cells accumulate β-catenin (white asterisk). Results are representative of experiments conducted in at least three different mice for each stage. Scale bars: 100 μm

The pituitary gland of the *Hesx1*^*Cre/+*^*;Ctnnb1*^*lox(ex3)/+*^*;R26*^*YFP/+*^ mice at 18.5 dpc was enlarged in comparison with the *Hesx1*^*Cre/+*^*;R26*^*YFP/+*^ controls, but did not develop tumours at this stage and will be hereafter referred to as a ‘pre-tumoural’ pituitary (Fig. 1a). The vast majority of the cells in the developing anterior pituitary were YFP+ with abundant Ki67 expression, as expected at this developmental stage (Fig. 1c). After a variable latency period of several weeks (median age of 17.7 weeks, range 8.3–35.3 weeks^37^), the YFP+ population was progressively replaced by actively proliferating YFP−;Ki67+ cells (Fig. 1b, c). Eventually, in fully developed tumours (i.e. when mice reached the humane end-point), none or only scarce YFP+ cells could be observed in the periphery of the majority of the tumours (8 out of 11), which were comprised mostly of YFP− cells. Notably, Ki67 expression was mostly observed in YFP− cells at this point (Fig. 1c). Similar results were obtained from *Hesx1*^*Cre/+*^*;Ctnnb1*^*lox(ex3)/+*^*;R26*^*mTmG/+*^ mice, in which 4 out of 5 tumours contained large tdTomato+ areas, indicating that tumour cells were not recombined (Supplementary Fig. 1). β-catenin-accumulating cell clusters were evident at pre-tumoural stages and as expected were YFP+ (Fig. 1c) and Ki67−^27^. As tumours developed, β-catenin expression was elevated in YFP− cells and clusters were no longer identifiable (e.g. after 20 weeks) (Fig. 1c). Additionally, YFP− cells were synaptophysin-, suggesting that these are not neuroendocrine cells (Fig. 1c).

Exome sequencing of 15 murine tumours revealed the presence of a total of 272 mutations (average 18.13 mutations per tumour; Supplementary Data 2) of which 131 were in coding regions (average 8.73 mutations per tumour; Supplementary Data 2). Of a selection of 14 of these 131 mutations, we validated 12 by Sanger sequencing (i.e. they were identified in the same DNA used for exome sequencing) (Supplementary Data 2). Only three genes were recurrently mutated in either two or three separate tumours and no recurrent mutations were found present in all 15 tumours (Supplementary Data 2). A low mutational burden with few recurrent mutated genes, other than *CTNNB1*, has been described in human ACP^33^. To determine if these changes are representative of the mutagenic process occurring in human ACP, we carried out comparisons with the published exome sequence results of 12 human ACPs^33^. This revealed 12 genes carrying mutations in both human and mouse tumours, but none of these mutations were identical between species (Supplementary Data 2). Five of these 12 mutated genes harboured non-silent/intronic mutations in both species (Table 1). The functional significance of these mutations is currently unknown.

**Table 1:** Recurrently mutated genes in mouse and human ACP

Human ACP				*Hesx1*^*Cre/+*^*;Ctnnb1*^*lox(ex3)/+*^ tumours			
Gene	cDNA change	Protein change	Variant classification	Gene	cDNA change	Protein change	Variant classification
*ASCC2*	c.1861G>A	p.D621N	Missense	*Ascc2*	c.1016+10G>A	NA	Splice region
*DNAJC3*	c.1243C>T	p.R415*	Nonsense	*Dnajc3*	c.1381G>T	p.G461*	Nonsense
*KIF1C*	c.2087G>A	p.R696Q	Missense	*Kif1c*	c.474G>T	p.L158F	Missense
*PKD1L1*	c.3862C>T	p.R1288C	Missense	*Pkd1l1*	c.55G>T	p.W19C	Missense
*TGFBI*	c.1286C>T	p.A429V	Missense	*Tgfbi*	c.662A>C	p.D221A	Missense

*Nonsense mutation

Together, these findings demonstrate that the activation of oncogenic β-catenin in Hesx1+ pituitary embryonic precursors leads to cell transformation and formation of somatic mutations-bearing tumours, which are mostly not derived from the *Hesx1* cell lineage. Additionally, our time course analysis reveals that tumour initiation requires a latency period of several weeks and is preceded by formation of β-catenin-accumulating cell clusters in the pre-tumoural pituitary.

### ACP clusters contain senescent cells with an activated SASP

The observation that cluster cells do not divide led us to hypothesise that the expression of mutant β-catenin may result in oncogene-induced senescence. We analysed the expression of a panel of senescence-associated markers by immunostaining in mutant mouse embryos at 18.5 dpc and 23 different human ACP samples to assess whether hallmark senescence features were present in cluster cells including: (i) cell cycle arrest; (ii) DNA damage and activation of a DDR and (iii) increased lysosomal content^1, 38^.

**Fig. 2:**
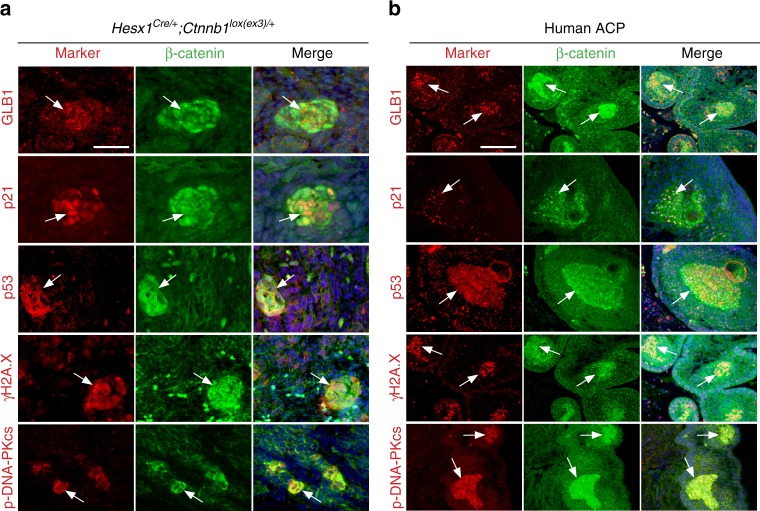
β-catenin-accumulating cluster cells in mouse and human ACP express senescence markers. Double immunostaining on histological sections of *Hesx1*^*Cre/+*^*;Ctnnb1*^*lox(ex3)/+*^ pre-tumoural pituitaries at 18.5 dpc (**a**) and human ACP tumours (**b**). Note that cluster cells (arrows) express lysosomal enzymes such as GLB1, encoding senescence-associated β-galactosidase, the cell cycle regulators p21 and p53, as well as the DNA damage and DNA damage response markers gamma histone 2AX and phospho-DNA-PKcs, respectively. Results are representative from experiments conducted in six to eight mice and 23 human ACP tumours. Normal brain was used as negative control. Scale bars: **a** 50 μm; **b** 100 μm

Cluster cells were negative for the mitotic marker phospho-histone H3 and did not incorporate 5-ethynyl-2′-deoxyuridine (EdU), a specific indicator of S phase. Cell death was not detected by caspase 3 immunostaining (Supplementary Fig. 2). Human β-catenin-accumulating cluster cells are also negative for Ki67 and caspase 3^36, 39^. Murine and human clusters exhibited marked expression of p53 (TP53), p21 (CDKN1A) and p16 (CDKN2A), all crucial effectors of cell cycle arrest^38^ (Fig. 2a, b; Supplementary Fig. 3a, b). Cluster cells in mouse and human ACP expressed γ-H2AX, suggesting the presence of DNA damage^40^; as well as phospho-DNA-PKcs, PARP1 and phospho-ATM, therefore revealing DDR activation^41, 42^ (Fig. 2a, b; Supplementary Fig. 3c, d). The senescence-associated β-galactosidase (SA-βgal) staining is a classical assay to identify senescent cells by detecting the presence of the lysosomal enzyme β-d-galactosidase^43^, the product of the *GLB1* gene^44^. SA-βgal staining on *Hesx1*^*Cre/+*^*;Ctnnb1*^*lox(ex3)/+*^ mutant pituitaries (Supplementary Fig. 3e) and double immunofluorescence for β-catenin and GLB1 revealed the expression of GLB1 in the cell clusters in both mouse and human ACP (Fig. 2a, b). LAMP1, LAMP2 and lysozyme C expression were also upregulated in cluster cells, suggesting the enlargement of the lysosomal compartment, another feature of senescent cells (Supplementary Fig. 3f, g)^44, 45^.

**Fig. 3:**
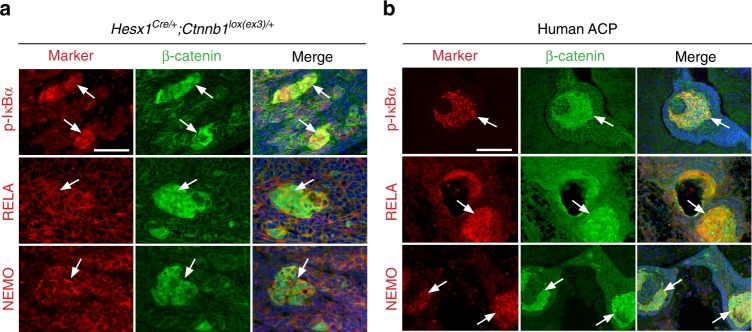
Cluster cells show activation of the NFκB pathway. Double immunostaining on histological sections of *Hesx1*^*Cre/+*^*;Ctnnb1*^*lox(ex3)/+*^ pre-tumoural pituitaries at 18.5 dpc (**a**) and human ACP tumours (**b**). Phospho-IκBα, a readout of activated NFκB pathway, as well as RELA and NEMO, two components of this pathway, are expressed in cluster cells (white arrows). Results are representative of experiments conducted in four to six mice. Scale bar: **a** 50 μm; **b** 100 μm

The activation of the NF-κB pathway is required for survival of senescent cells and activation of SASP^11^. As a read out of an activated NF-κB pathway, we assessed the presence of phospho-IκBα, as phosphorylation at Ser32 and Ser36 of IκBα is followed by proteasome-mediated degradation and pathway activation^46^. Mouse and human clusters expressed high levels of p-IκBα (Fig. 3a, b). RELA/p65 and NEMO, two crucial components of the NF-κB pathway, were broadly expressed throughout the tissue, including cluster cells of both species (Fig. 3a, b). Finally, to determine if senescence and the SASP response are initiated together with cluster formation and prior to any tumour initiation, we turned to the early stages of mutant pituitary development in the embryonic mouse model. Analysis of *Hesx1*^*Cre/+*^*;Ctnnb1*^*lox(ex3)/+*^ embryonic pituitaries from 10.5 to 18.5 dpc revealed a dynamic response for the different aspects of senescence in the cluster cells. Proliferation in β-catenin-accumulating cluster cells, as assessed by Ki67 and EdU incorporation, was higher at 10.5 dpc than 18.5 dpc, whilst expression of the cell cycle inhibitors p16 and p21 was detected in the majority of the cluster cells from 10.5 dpc (Supplementary Fig. 4a, b). The DDR and NF-κB pathways became active by 10.5–12.5 dpc (Supplementary Fig. 4c), whereas expansion of the lysosomal compartment occurred later in development around 16.5–18.5 dpc (Supplementary Fig. 4a, b, d). Therefore, the senescence/SASP response is initiated at early stages of development, soon after the emergence of tumours.

**Fig. 4:**
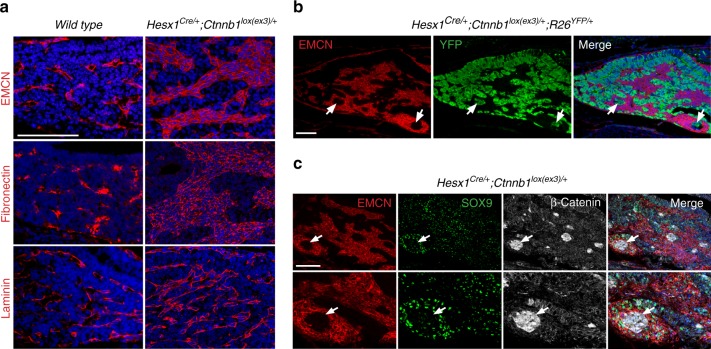
Tissue microenvironment changes in the pre-tumoural pituitary of *Hesx1*^*Cre/+*^*; Ctnnb1*^*lox(ex3)/+*^ mutants. **a** Immunostaining against endomucin (EMCN), fibronectin and laminin revealing changes in the parenchyma of the pre-tumoural pituitary at 18.5 dpc. Scale bar: 100 μm. **b** Mutually exclusive staining of YFP+ (detected with an anti-GFP antibody) and EMCN+ cells. Note the presence of YFP+ cells (arrows) surrounding a population of EMCN+ cells. Scale bar: 100 μm. **c** Triple immunostaining showing the co-expression of EMCN and SOX9 in large group of cells around the β-catenin-accumulating clusters (arrows). Results are representative of experiments conducted in six mice. Scale bar: 100 μm

SASP activation results in alterations in normal tissue architecture and increased angiogenesis^6^, therefore, we sought to analyse the tumour microenvironment in *Hesx1*^*Cre/+*^*;Ctnnb1*^*lox(ex3)/+*^ pituitaries at 18.5 dpc. In controls, immunostaining against endomucin (EMCN), a sialoglycoprotein routinely used as a marker of endothelial cells^47^, revealed the presence of cells with a thin and elongated morphology surrounding the lumen of blood vessels in the anterior lobe of the control pituitaries (Fig. 4a). In contrast, *Hesx1*^*Cre/+*^*;Ctnnb1*^*lox(ex3)/+*^ pituitaries displayed a drastic expansion of the EMCN+ population, which formed large and dense groups of cells without the presence of a lumen (Fig. 4a). Double immunostaining against YFP and EMCN in the *Hesx1*^*Cre/+*^*;Ctnnb1*^*lox(ex3)/+*^*;R26*^*YFP/+*^ pituitaries revealed mutually exclusive staining with the EMCN cells occupying almost entirely the YFP− areas (Fig. 4b). Expression of extra-cellular matrix proteins such as fibronectin and laminin was altered in the *Hesx1*^*Cre/+*^*;Ctnnb1*^*lox(ex3)/+*^ pre-tumoural pituitaries when compared with controls (Fig. 4a). Interestingly, EMCN+ cells frequently formed ring-like structures surrounding YFP+ cells, which resembled the β-catenin-accumulating clusters. Triple immunostaining against SOX9, β-catenin and EMCN confirmed that the cells directly surrounding the clusters are SOX9+;EMCN+ (Fig. 4c). Together, these results suggest that cluster cells activate a senescence and SASP programme leading to changes in the cellular microenvironment of the pre-tumoural pituitary.

### ACP clusters show a senescence/SASP molecular signature

The expression of oncogenic β-catenin in Sox2+ pituitary stem cells in *Sox2*^*CreERT2/+*^*;Ctnnb1*^*lox(ex3)/+*^*;R26*^*YFP/+*^ mice at 4–6 weeks of age results in cluster formation and induction of paracrine tumours^28^. However, the molecular signature of the cluster cells in *Sox2*^*CreERT2/+*^*;Ctnnb1*^*lox(ex3)/+*^*;R26*^*YFP/+*^ mice has not yet been revealed. To this end, we first induced *Sox2*^*CreERT2/+*^*;Ctnnb1*^*lox(ex3)/+*^;*R26*^*YFP/+*^ mice at 4 weeks of age by tamoxifen administration and analysed the pituitaries 4 weeks later. Following dissociation into single-cell suspensions, YFP+ and YFP− cells were isolated by flow sorting and subjected to qRT-PCR analysis. This revealed a gene expression profile consistent with the presence of β-catenin-accumulating cluster cells in the YFP+ population (Supplementary Fig. 5b).

Next we sought to reveal the molecular signature of cluster cells relative to normal Sox2+ cells not expressing oncogenic β-catenin. *Sox2*^*CreERT2/+*^*;Ctnnb1*^*lox(ex3)/+*^;*R26*^*YFP/+*^ mutant and *Sox2*^*CreERT2/+*^*;R26*^*YFP/+*^ control mice were tamoxifen induced at 4 weeks of age and pituitaries dissociated and subjected to RNA sequencing 4 weeks later. The expression data for all of the 27,179 genes are included in Supplementary Data 3 and the demonstration of a successful experimental approach in Supplementary Fig. 5a. Of note, differential expression studies demonstrated the upregulation of several members of the hedgehog (HH), fibroblast growth factor (FGF), bone morphogenetic protein (BMP), wingless-related integration site (WNT) and transforming growth factor β (TGFβ) families as well as CXC chemokines and interleukins (Supplementary Data 3). These results support the notion that cluster cells are signalling hubs in the pre-tumoural pituitary.

**Fig. 5:**
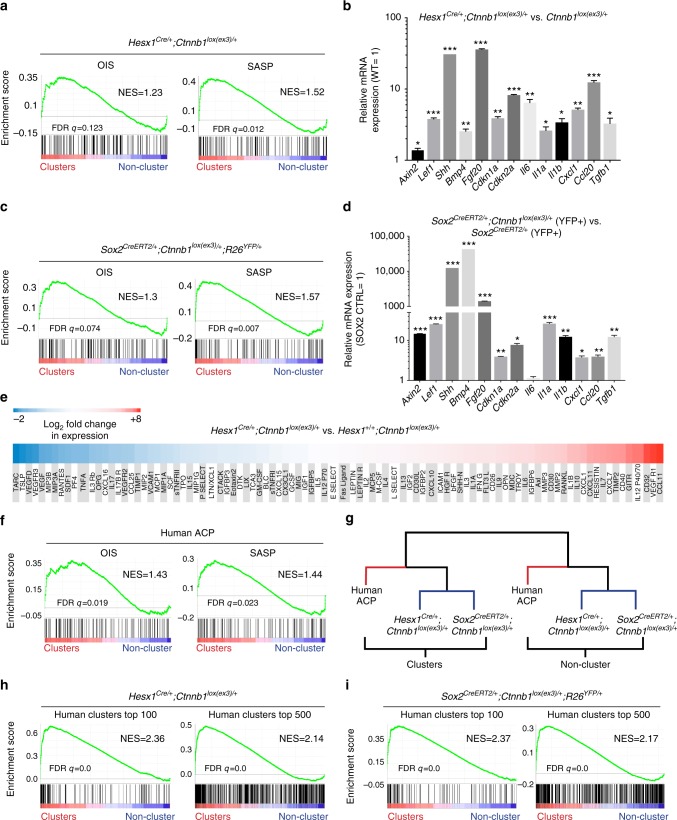
Mouse and human β-catenin-accumulating clusters share a common signature of senescence and SASP. **a**–**d** Gene set enrichment analysis (GSEA) (**a**, **c**) revealing that clusters from both mouse models, the *Hesx1*^*Cre/+*^*;Ctnnb1*^*lox(ex3)/+*^ and *Sox2*^*CrERT2e/+*^*;Ctnnb1*^*lox(ex3)/+*^*;R26*^*YFP/+*^, show a molecular signature of oncogene-induced senescence (OIS) and senescence-associated secretory phenotype (SASP). qRT-PCR analysis showing the upregulation of several senescence and SASP factors in both mouse models (**b**, **d**). Bars in **b** and **d** represent the mean, and error bars represent standard error of the mean (SEM) of three biological replicates. *p < 0.05, **p < 0.01, ***p < 0.001, Student’s *t* test. **e** Several cytokines are upregulated in the *Hesx1*^*Cre/+*^*;Ctnnb1*^*lox(ex3)/+*^ mutant relative to the *Hesx1*^*+/+*^*;Ctnnb1*^*lox(ex3)/+*^ control pituitaries as measured in a mouse cytokine antibody array. **f** Gene set enrichment analysis (GSEA) demonstrating that laser-capture micro-dissected human clusters show a molecular signature of oncogene-induced senescence (OIS) and senescence-associated secretory phenotype (SASP). **g** Hierarchical clustering analysis of RNA-data sets from cluster and non-cluster cell populations indicating coherent grouping between specific cell compartments. **h**, **i** GSEA of the top 100 or 500 most upregulated genes in human clusters and the whole transcriptome of mouse clusters from both models demonstrating a common molecular signature

To assess in an unbiased manner whether cluster cells in *Sox2*^*CreERT2/+*^*;Ctnnb1*^*lox(ex3)/+*^ and *Hesx1*^*Cre/+*^*;Ctnnb1*^*lox(ex3)/+*^(previously characterised^48^) mice activate senescence and a SASP, we specifically compared the expression profiles of the murine clusters with established molecular signatures for OIS and SASP obtained from human IMR90 ER:RAS fibroblasts. These cells express a Steroid Receptor-KRAS^G12V^ chimaeric fusion protein that triggers growth arrest, senescence and SASP^13^. Gene set enrichment analysis (GSEA) showed that the gene expression profiles of the clusters in both mouse models were significantly enriched for OIS and SASP genes (Fig. 5a, c). This was further validated by qRT-PCR (Fig. 5b, d). SASP activation was further investigated using semi-quantitative ELISA cytokine arrays, which demonstrated the upregulation of several SASP-related proteins, such as ICAM1, bFGF, IL1A, IFNG, OPN, IL6, IGFBL6, MMP3, MMP2, RNAKL1, CXCL1, CXCL11, IL7, TIMP2, VEGFR1 and CCL11) (Fig. 5e).

To determine whether these findings were relevant to human ACP, we assessed the degree of molecular equivalence between mouse and human ACP clusters. We recently analysed the molecular signatures of specific tumour cell compartments in human ACP including the β-catenin-accumulating cell clusters, by combining laser-capture microdissection and RNA-Sequencing (J.R. Apps and J.P. Martinez-Barbera, manuscript submitted, Array Express accession code: E-MTAB-5266). Hierarchical clustering analysis revealed the grouping of *Hesx1*^*Cre/+*^*;Ctnnb1*^*lox(ex3)/+*^, *Sox2*^*CreERT2/+*^*;Ctnnb1*^*lox(ex3)/+*^ and human clusters, suggesting similar molecular profiles (Fig. 5g), while GSEA confirmed that human clusters also have a molecular signature of senescence and SASP (Fig. 5f). Moreover, GSEA analysis of *Hesx1*^*Cre/+*^*;Ctnnb1*^*lox(ex3)/+*^ and *Sox2*^*CreERT2/+*^*;Ctnnb1*^*lox(ex3)/+*^ cluster gene expression profiles showed a significant enrichment for the top 100 and 500 most upregulated genes in human clusters (Fig. 5h, i). Together, these analyses support that human and mouse clusters are equivalent structures, sharing a common signature of senescence and SASP.

### Aged Sox2+ stem cells show reduced tumour-inducing potential

We sought to determine whether the age at which *Sox2*^*CreERT2/+*^*;Ctnnb1*^*lox(ex3)/+*^ mice were targeted to express the oncogenic β-catenin may have an effect on paracrine tumourigenesis.

*Sox2*^*CreERT2/+*^*;Ctnnb1*^*lox(ex3)/+*^ mutant mice were tamoxifen induced at either 1 month, when pituitary stem cells are still actively supporting organ growth, or at 6–9 months of age when contribution from Sox2+ stem cells to homeostasis is low. Pituitaries were analysed at 3 months post induction and assessed for tumour formation. Histological analysis revealed that 15/15 mice that were induced at 1 month of age developed pituitary tumours with a total of 48 individual lesions (average 2–4 tumours per pituitary). However, only 3 out of 17 mice induced at 6–9 months of age developed pituitary tumours with only five individual lesions observed (*p* < 0.0001; Table 2).

**Table 2:** Reduced tumour-inducing potential in older vs. young Sox2+ pituitary stem cells

*Sox2* ^*CreERT2/+*^ *;* *Ctnnb1* ^*lox(ex3)/+*^ *;* *R26* ^*YFP/+*^		
Age at tamoxifen induction	Tumour incidence^a^	Number of mice
1 month	15 (48 lesions)	15
6–9 months*	3 (5 lesions)	17

*Unpaired *t*-test showed a statistically significant difference in tumour formation between age groups (*p* = < 0.0001)

^a^ Tumour incidence was assessed histologically at 3 months post-tamoxifen induction

**Fig. 6:**
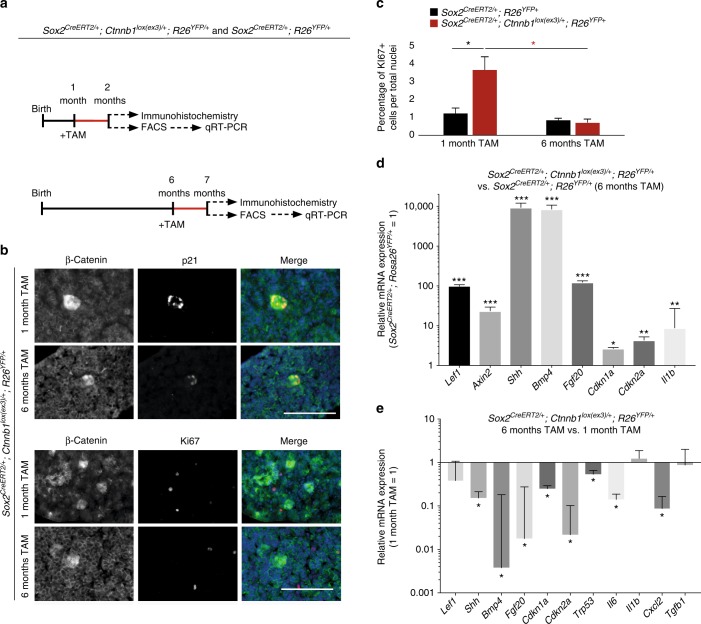
Aged Sox2+ stem cells generate β-catenin-accumulating cell clusters with reduced senescence and SASP responses. **a** Schematic diagram of the experimental designs. *Sox2*^*CreERT2/+*^*;Ctnnb1*^*lox(ex3)/+*^*;R26*^*YFP/+*^ and *Sox2*^*CreERT2/+*^*;R26*^*YFP/+*^ mice were tamoxifen induced at either 1 or 6 month of age and the anterior pituitary collected 4 weeks post induction and processed for histological and molecular analysis of YFP+ cells isolated by FACS (*n* = 8 mice per group). **b** Double immunostaining on histological sections of *Sox2*^*CreERT2/+*^*; Ctnnb1*^*lox(ex3)/+*^*; R26*^*YFP/+*^ pituitaries revealing the presence of β-catenin-accumulating cell clusters positive for p21, but not for Ki67. Scale bar: 100 µm. **c** Quantitative analysis showing a significant higher Ki67 proliferation index in non-cluster cells in *Sox2*^*CreERT2/+*^*;Ctnnb1*^*lox(ex3)/+*^*;R26*^*YFP/+*^ tumoural relative to *Sox2*^*CreERT2/+*^*;R26*^*YFP/+*^ control pituitaries in tamoxifen-induced mice at 1 month (black asterisk), but not at 6 months. Increased proliferation index is also observed in non-cluster cells in *Sox2*^*CreERT2/+*^*;Ctnnb1*^*lox(ex3)/+*^*;R26*^*YFP/+*^ pituitaries of mice induced at 1 month relative to those induced at 6 months (red asterisk). **d** qRT-PCR analysis showing the upregulation of the WNT pathway target genes *Axin2* and *Lef1*, as well as of several senescence and SASP factors in the *Sox2*^*CreERT2/+*^*;Ctnnb1*^*lox(ex3)/+*^*;R26*^*YFP/+*^ mutant pituitaries relative to the *Sox2*^*CreERT2/+*^*; R26*^*YFP/+*^ controls. **e** qRT analysis showing the relative expression of several senescence and SASP factors in *Sox2*^*CreERT2/+*^*;Ctnnb1*^*lox(ex3)/+*^*;R26*^*YFP/+*^ mice tamoxifen induced at either 1 (young) or 6 (older) months of age. The expression of several senescent and SASP factors is significantly reduced in the older compared with the young mice. Bars in **c**, **d** and **e** represent the mean, and error bars represent standard error of the mean (SEM) of three biological replicates. *p < 0.05, **p < 0.01, ***p < 0.001, Student’s *t* test

We decided to explore whether the reduced tumour burden in older *Sox2*^*CreERT2/+*^*;Ctnnb1*^*lox(ex3)/+*^ mice may be related to an attenuated senescence and SASP response in the β-catenin-accumulating clusters. To achieve this, YFP+ cells were isolated by flow cytometry from *Sox2*^*CreERT2/+*^*;Ctnnb1*^*lox(ex3)/+*^*;R26*^*YFP/+*^ mutant and *Sox2*^*CreERT2/+*^*;R26*^*YFP/+*^ control mice that were induced at either one month or 6–9 months of age (Fig. 6a). Relative to age-matched control YFP+ cells, YFP+ cells from older mice activated the WNT pathway and a senescence/SASP programme (Fig. 6d). Although both age groups contained Ki67−/p21+ β-catenin-accumulating clusters (Fig. 6b), the average number of cells in the clusters of young-induced *Sox2*^*CreERT2/+*^*;Ctnnb1*^*lox(ex3)/+*^*;R26*^*YFP/+*^ mutant mice was 3.16 ± 1.57 vs. 1.95 ± 1.12 in older-induced animals, affirming that clusters from older mice were smaller in size (*p* < 0.0001, Student’s *t* test; 57 clusters counted in three young *Sox2*^*CreERT2/+*^*;Ctnnb1*^*lox(ex3)/+*^*;R26*^*YFP/+*^ pituitaries and 94 clusters counted in six older *Sox2*^*CreERT2/+*^*;Ctnnb1*^*lox(ex3)/+*^*;R26*^*YFP/+*^ pituitaries). In addition, increased Ki67 index was observed only in non-cluster cells in the younger, but not in the older *Sox2*^*CreERT2/+*^*;Ctnnb1*^*lox(ex3)/+*^*;R26*^*YFP/+*^ pituitaries, suggesting that Sox2+ cluster cells from younger mice can induce a proliferative response in surrounding cells (Fig. 6c).

Importantly, comparative analysis between the YFP+ fractions of *Sox2*^*CreERT2/+*^*;Ctnnb1*^*lox(ex3)/+*^*;R26*^*YFP/+*^ mutants revealed a significant reduction in the expression of senescence and SASP factors in mice induced at 6–9 months relative to the one month-induced mice (Fig. 6e). We also observed an age-dependent increase in the expression of senescence-associated markers, such as p16 (*Cdkn2a*) and p53 (*Trp53*), in non-mutant Sox2+ cells in *Sox2*^*CreERT2/+*^*;R26*^*YFP/+*^ control pituitaries, which may underlie the ameliorated senescence/SASP response of older relative to younger mutant Sox2+ stem cells (Supplementary Fig. 6). Together, these analyses demonstrate that expression of oncogenic β-catenin in Sox2+ stem cells in 6–9 months old mice is less tumourigenic and leads to weakened senescence and SASP responses with formation of smaller β-catenin-accumulating clusters.

### *Apc*-deficient clusters lack tumour-inducing potential

The data previously described in *Hesx1*^*Cre/+*^*;Ctnnb1*^*lox(ex3)/+*^ and *Sox2*^*CreERT2/+*^*;Ctnnb1*^*lox(ex3)/+*^ mice are compatible with a model whereby senescent cells in the β-catenin-accumulating clusters of the pre-tumoural pituitary may initiate tumourigenesis by activating a robust SASP programme. Therefore, we sought to explore whether the reduction or ablation of senescent cells may prevent or delay tumourigenesis.

The senolytic compounds ABT-263 and ABT-737 have been shown to selectively ablate senescent cells in vitro and in vivo^49, 50^. In agreement with these findings, ex vivo culture of 18.5 dpc *Hesx1*^*Cre/+*^*;Ctnnb1*^*lox(ex3)/+*^ pre-tumoural pituitaries in the presence of ABT-263 and ABT-737 revealed a reduction in the average size of the clusters, but this did not reach significance (number of cells per cluster: vehicle 2.46 ± 1.26, 41 clusters counted from three pituitaries; ABT-263, 1.4 ± 0.49, *p* = 0.47, 40 clusters counted from four pituitaries; ABT-737, 1.8 ± 0.62, *p* = 0.70, 38 clusters counted from three pituitaries; Student’s *t* test) (Supplementary Fig. 7c, d). In contrast, treatment of IMR90 ER:RAS senescent fibroblasts with these two senolytic agents significantly increased cell death relative to control non-senescent cells (Supplementary Fig. 7a, b). The distinct target cell type (i.e. epithelial cluster cell rather than fibroblast) may confer a resistance to these drugs and, additionally, drug delivery and dose may require further optimisation. The drugs target genes *Bcl2*, *Bcl-xl* and *Bcl-w* are all expressed in the cluster cells, but not upregulated.

Since chemical ablation of senescent cells was not strong enough to reduce significantly the size of the clusters, we sought to explore whether a genetic approach may impair the senescent/SASP response more robustly. It has been shown that the strength of the senescence/SASP response depends on the oncogenic hit; e.g. the activation of the mitogen-activated protein kinase (MAPK) pathway by BRAF^V600E^ is a more potent senescence inducer than KRAS^G12V^ ^51^. We reasoned that the deletion of tumour suppressor *Apc* in *Hesx1*^*Cre/+*^*;Apc*^*fl/fl*^ mice would lead to the activation of the WNT pathway in embryonic precursors and the accumulation of wild-type β-catenin rather than its degradation-resistant form, which is encoded by the oncogenic *Ctnnb1* locus in *Hesx1*^*Cre/+*^*;Ctnnb1*^*lox(ex3)/+*^ mice. We expected wild-type β-catenin to be a weaker senescence inducer than oncogenic β-catenin, resulting in reduced senescence/SASP activation.

**Fig. 7:**
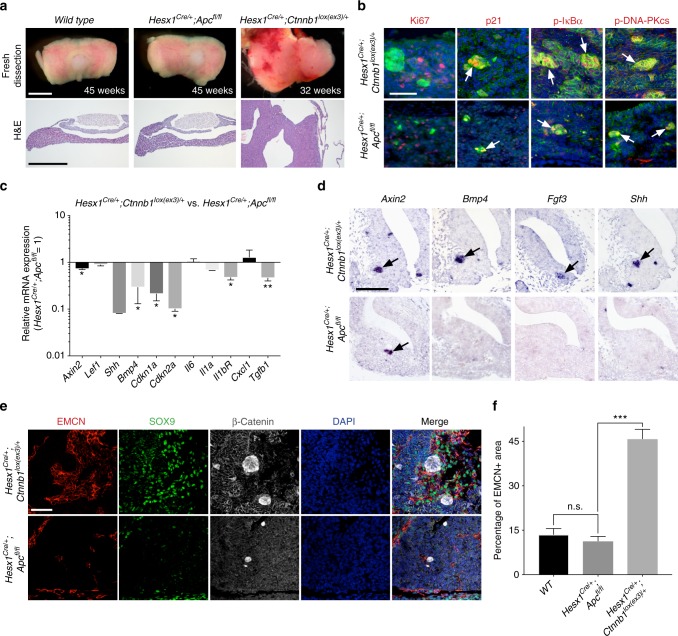
Deletion of the tumour suppressor *Apc* results in β-catenin-accumulating senescent clusters with reduced SASP. **a** Brightfield and haematoxylin–eosin pictures of wild-type and *Hesx1*^*Cre/+*^*;Apc*^*fl/fl*^ pituitaries, and *Hesx1*^*Cre/+*^*;Ctnnb1*^*lox(ex3)/+*^ pituitary tumours. Note that the *Apc*-deficient pituitary is enlarged but no tumours are apparent and it is histologically normal. The age of the mice is indicated in weeks. Scale bar: 1 mm. **b** Double immunostaining revealing the expression of p21, phospho-IκBα and phospho-DNA-PKcs in the β-catenin-accumulating clusters, indicating cell cycle inhibition and activation of the NFκB and DNA damage response pathways, in both *Hesx1*^*Cre/+*^*;Apc*^*fl/fl*^ and *Hesx1*^*Cre/+*^*;Ctnnb1*^*lox(ex3)/+*^ pituitaries at 18.5 dpc. Note that the clusters are smaller in the *Hesx1*^*Cre/+*^*;Apc*^*fl/fl*^ compared with the *Hesx1*^*Cre/+*^*;Ctnnb1*^*lox(ex3)/+*^ pituitaries. Scale bar: 50 μm. **c** qRT-PCR analysis showing the relative expression of several genes in *Hesx1*^*Cre/+*^*;Apc*^*fl/fl*^ relative to *Hesx1*^*Cre/+*^*; Ctnnb1*^*lox(ex3)/+*^ pituitaries at 18.5 dpc. The expression of the WNT pathway targets *Axin2* and *Lef1* are similar between the two genotypes, but several senescence and SASP factors show reduced expression levels in the *Hesx1*^*Cre/+*^*;Apc*^*fl/fl*^ compared with the *Hesx1*^*Cre/+*^*;Ctnnb1*^*lox(ex3)/+*^ pituitaries. Bars represent the mean, and error bars represent standard error of the mean (SEM) of three biological replicates. *p < 0.05, **p < 0.01, Student’s *t* test. **d** In situ hybridisation on histological sections of embryos at 14.5 dpc showing the expression of *Axin2* in small cell clusters and very reduced or absent detection of *Bmp4*, *Fgf3* and *Shh* in the *Apc*-deficient pituitaries. Scale bar: 100 μm. **e** Triple immunostaining revealing the expression of β-catenin, SOX9 and EMCN. Note that the large areas of EMCN+;SOX9+ cells surrounding the β-catenin-accumulating clusters are not present in the *Hesx1*^*Cre/+*^*;Apc*^*fl/fl*^ pituitaries. Scale bar: 50 μm. **f** Quantification of the EMCN+ areas demonstrating that wild-type and *Apc*-deficient pituitaries show similar levels, which are significantly increased in the *Hesx1*^*Cre/+*^*;Ctnnb1*^*lox(ex3)/+*^ pituitaries. Bars represent the mean, and error bars represent standard error of the mean (SEM) of three biological replicates. ***p < 0.001, one-way ANOVA. All results are representative of at least three biological replicates

*Hesx1*^*Cre/+;*^*Apc*^*fl/fl*^ mice were born at the expected Mendelian ratios, were normal and fertile with proper specification of pituitary endocrine lineages (Supplementary Data 4**;** Supplementary Fig. 8a). Although slight pituitary hyperplasia was consistently observed, no pituitary tumours ever formed in *Hesx1*^*Cre/+;*^*Apc*^*fl/fl*^ mice (*n* = 61), even when followed until 18 months of age (*n* = 17) (Fig. 7a; Supplementary Fig. 8a). Immunostaining studies revealed the presence of nucleo-cytoplasmic β-catenin-accumulating clusters in *Hesx1*^*Cre/+;*^*Apc*^*fl/fl*^ embryos. These clusters resembled their oncogenic β-catenin counterparts in many ways, such as in the expression of SOX2, but not SOX9, and WNT pathway activation as evidenced by the over-expression of the WNT-target gene *Axin2* (Fig. 7d; Supplementary Fig. 8b). Additionally, cluster cells in the *Apc* mutant pituitary were negative for Ki67, phospho-Histone H3, or Caspase 3 and expressed the cell cycle inhibitors p21 and p16 (Fig. 7b; Supplementary Fig. 8c, d). Moreover, these clusters were strongly positive for DDR and NF-κB activation markers such as phospho-DNA-PKcs, PARP-1 and phospho-IκBα, indicative of the activation of a senescence programme (Fig. 7b; Supplementary Fig. 8d). Despite the similarities in gene expression, the average cluster size at 18.5 dpc in *Hesx1*^*Cre/+;*^*Apc*^*fl/fl*^ pituitaries was 1.97 ± 1.36 cells, rather than 5.89 ± 0.33 in *Hesx1*^*Cre/+*^*;Ctnnb1*^*lox(ex3)/+*^ embryos (*p* < 0.0001, Student’s *t* test; 126 clusters counted for *Hesx1*^*Cre/+*^*;Ctnnb1*^*lox(ex3)/+*^ and 108 clusters counted for *Hesx1*^*Cre/+;*^*Apc*^*fl/fl*^ in three different pituitaries).

qRT-PCR analysis comparing *Hesx1*^*Cre/+;*^*Apc*^*fl/fl*^ to *Apc*^*fl/+*^ control pituitaries revealed no significant differences in the expression levels of several senescence and SASP factors including *Cdkn1a*, *Cdkn2a*, *Shh*, *Bmp4*, *Il1a*, *Il1b*, *Il6*, *Cxcl1* and *Tgfb1* (Supplementary Fig. 8e). However when comparing expression levels of senescence/SASP factors relative to *Hesx1*^*Cre/+;*^*Ctnnb1*^*lox(ex3)/+*^ pituitaries, these were reduced in *Hesx1*^*Cre/+*^*;Apc*^*fl/fl*^ mutants (Fig. 7c), suggesting an overall reduction of the senescence/SASP response upon *Apc* deletion and in line with the formation of smaller clusters. In situ hybridisation previously confirmed the upregulation of *Axin2*, *Bmp4*, *Fgf3* and *Shh* in *Hesx1*^*Cre/+;*^*Ctnnb1*^*lox(ex3)/+*^ clusters^27^. In contrast, despite *Hesx1*^*Cre/+*^*;Apc*^*fl/fl*^ clusters expressing *Axin2* and demonstrating WNT pathway activation, the expression of *Bmp4*, *Fgf3* and *Shh* expression was not detectable (Fig. 7d). Moreover, the abundant SOX9+;EMCN+ cells surrounding the clusters in the *Hesx1*^*Cre/+*^*;Ctnnb1*^*lox(ex3)/+*^ pituitaries (Fig. 4c) were not apparent in *Hesx1*^*Cre/+*^*;Apc*^*fl/fl*^ mutant glands (Fig. 7e), and overall quantification of the area covered by endomucin positive cells revealed values comparable to control littermates (Fig. 7f). In a *Hesx1*^*Cre/+*^*;Apc*^*fl/fl*^ pituitary where a larger cluster was identified, accumulation of EMCN+ cells was observed around it (Supplementary Fig. 8f), suggesting that a threshold of SASP factors may be needed to elicit the changes in tissue architecture. Finally, no tumours developed in *Sox2*^*CreERT2/+*^*;Apc*^*fl/fl*^ mice induced from 4 to 8 weeks of age in a follow up of up to a year (*n* = 7 mice). Together, these studies demonstrate that *Apc* deletion in pituitary embryonic precursors/stem cells leads to reduced senescence/SASP response and is not tumorigenic.

## Discussion

**Fig. 8:**
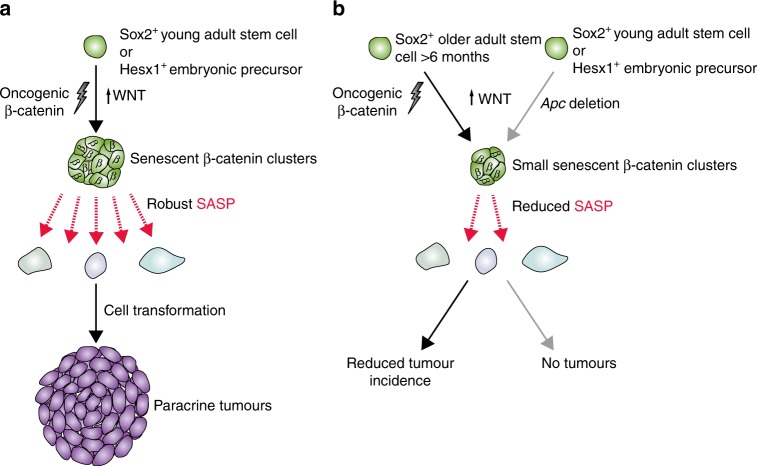
Schematic summary of the main findings of this research. **a** Targeting Sox2+ stem cells from young adults, or Hesx1+ embryonic precursors to express oncogenic β-catenin, results in formation of senescent cell clusters and a robust SASP. This leads to cell transformation and generation of the cell-of-origin of the paracrine tumours, thus the tumours are not derived from the mutation-sustaining cells. **b** In contrast, the expression of oncogenic β-catenin in Sox2+ stem cells of older mice leads to a reduced tumour incidence or the failure to produce tumours. In addition, upregulation of the WNT pathway through the deletion of *Apc* in young adult stem cells or embryonic precursors also fails to induce tumours. In all cases described in **b**, there is attenuated senescence and SASP activation

In this paper, we show that the expression of oncogenic β-catenin and activation of the WNT pathway in Hesx1+ embryonic pituitary precursors or Sox2+ pituitary stem cells in young mice results in the activation of a robust SASP in targeted cells and the subsequent paracrine formation of tumours. These mouse tumours carry a low burden of somatic mutations that are mostly non-recurrent, which overlap with human ACP. To our knowledge, this is the first time that SASP activation is associated with non-cell autonomous cell transformation (i.e. induction of somatic mutations) and tumour initiation in an in vivo murine model of a human tumour. Of note, we refer to cell transformation to indicate the occurrence of somatic mutations not present in the germline and the acquisition of a proliferative advantage of the tumour relative to the non-tumour cells. However, mouse and human ACP do not harbour mutations in critical cancer genes (e.g. *p53*, *p16*, *Myc*, *Braf*, *Egfr* among others) suggesting that these tumours are not fully transformed. This is not surprising as human ACP is a benign tumour that very rarely progresses into a malignancy. A summary of the main findings of this study is shown in Fig. 8.

In the embryonic mouse ACP model, tumour progression does not start immediately after birth, but only after a latency period in which the pre-tumoural pituitary contains senescent clusters with an activated SASP. This non-linear growth dynamics have recently been characterised by magnetic resonance imaging^37^. The pre-tumoural pituitary also shows abnormal tissue architecture and overall slow cell proliferation. However, once rapidly dividing tumour cells appear, the clusters and most of the normal pituitary tissue are displaced. This growth pattern is compatible with the clusters’ activities being required to initiate a process eventually leading to tumour initiation (hit-and-run model), rather than promoting tumour progression, which may be self-sustaining. Recently, it has been shown that senescence cells through SASP can induce dedifferentiation and stem cell marker expression^52^. Transcriptomics failed to reveal the expression of *Hesx1*, but stem cell markers such as SOX9, *Klf2*, *Klf4* and *Cmyc* were upregulated in mouse tumours (J.R. Apps and J.P. Martinez-Barbera, manuscript submitted), sometimes in close proximity to the senescent cluster cells (i.e. SOX9; Fig. 4c)^27^. Immunostaining against SOX2 showed lack of expression of this stem cell marker in the murine tumours (Supplementary Fig. 9). In human ACP, the expression of stem cell markers (e.g. SOX2, SOX9, KLF4 and OCT4) has been found to be upregulated^53^. These results are compatible with a potential role of the senescent cluster cells in the promotion of reprogramming in vivo, a hypothesis that merits further research.

Elegant genetic experiments have shown that senescence is important to prevent tumourigenesis in mouse models of pituitary adenoma^54^. In agreement with this notion, our data suggest that senescence has an early cell-autonomous function that prevents the expansion of cluster cells harbouring oncogenic β-catenin. However, we reveal an additional non-cell autonomous role of senescent cells, mediated by the persistent activation of SASP resulting in paracrine cell transformation and tumourigenesis. Ablation of the senescent cluster cells at specific time points using chemical or genetic approaches will further help dissect their role in tumourigenesis. In particular, it will help determine when and for how long their activities are required for tumour initiation and/or maintenance.

We also reveal molecular similarities between the mouse and human clusters, which provide insights into the pathogenesis of human ACP. In contrast to the mouse tumours, the β-catenin-accumulating clusters are present in fully developed human ACP and tend to be located at the tips of finger-like epithelial protrusions that invade the brain^36, 37^. Analysis of a human ACP xenograft model suggest that the activities of the clusters are important in orchestrating cell migration and tumour invasion^36^. We show that β-catenin clusters undergo senescence and initiate SASP during embryonic pituitary development in the mouse, and this supports the notion that childhood-onset ACP is a developmental tumour. This proposition is substantiated by the scrutiny of clinical data, which has revealed that paediatric ACP patients suffer significant height deficits around 12 months of age, many years before ACP diagnosis, suggesting potential growth hormone deficiency^55^. Of note, somatotrophs, which express growth hormone, are clearly defective in the *Hesx1*^*Cre/+;*^*Ctnnb1*^*lox(ex3)/+*^ ACP model by the end of gestation^27^. Therefore, clusters may develop embryonically in human patients and be present in the pituitary gland of the newborn prior to the appearance of any tumour mass. In fact, foetal ACPs displaying nucleo-cytoplasmic β-catenin-accumulating cells have recently been diagnosed in human foetus at 18 weeks^56^. Additionally, we show that the SASP response is attenuated with age in Sox2+ pituitary stem cells in older mice, which is in agreement with ACP being primarily a paediatric tumour^29^.

Paracrine tumourigenesis has been reported in other tumours and cancers, but so far not associated with senescence and SASP. Over-expression of platelet-derived growth factor (PDGF) (also a SASP factor) in neural precursors in mice and rats is sufficient to recruit normal neural stem and progenitor cells that acquired genetic aberrations and become malignant, capable of generating tumours upon transplantation^57–59^. Likewise, conditional deletion of *Notch1* in mouse skin epidermis^60^ or activation of β-catenin in hair follicles stem cells by deleting *Ctnnb1* exon 3^61^ leads to over-activation of the WNT/β-catenin pathway and induction of tumours/growths in a non-cell autonomous manner. Additionally, the expression of a constitutively active form of MEK1, activating the ERK MAPK pathway, in mouse epidermis results in skin polyps, which are mostly formed by cells not carrying oncogenic MEK1^62^. More strikingly, in a mouse model for leukaemia the expression oncogenic β-catenin (i.e. *Ctnnb1* deletion of exon3) in osteoblast precursors is sufficient to generate oncogenic hematopoietic stem cells capable of giving rise and propagating acute myeloid leukaemia^63^. Whether in these models the tumour-inducing cells are senescent and have activated SASP is an important aspect that merits further research. Our results support the possibility that the activities of the senescent cells result in changes in the cellular microenvironment driving genetic and possibly, epigenetic changes in neighbouring cells, which may fuel tumour initiation in some of these neoplasias.

In summary, this study provides evidence for a mechanism by which embryonic progenitors and postnatal stem cells can contribute to tumourigenesis in a non-cell autonomous manner, identifying senescence and SASP as mediators of this tumour-inducing potential. Recent research has demonstrated that clearance of senescent cells, either naturally occurring in aged mice or diseased related, extends lifespan, improves the physiological function of several organs and delays tumourigenesis^49, 50, 64^. Although the ablation of the cluster cells in the ACP mouse model was only partial, these results reinforce the idea that cluster cells are senescent and suggest that senolytic compounds may be of potential therapeutic interest against human ACP. The development of more specific senolytics capable of targeting a variety of senescent cells, including pituitary epithelial cells targeted with oncogenic β-catenin, will be required to further study the potential anti-tumourigenic effects of senescence ablation in the context of ACP and other tumours. In addition, further research is required to identify the pro- and anti-tumourigenic mediators in the SASP, and the mouse models presented here are suitable tools to dissect these opposite functions.

## Methods

### Mice and human samples of ACP

The *Ctnnb1*^*lox(ex3)/+*^, *Sox2*^*CreERT2/+*^, *Hesx1*^*Cre/+*^, *ROSA26*^*flox-stop-YFP/+*^, *Apc*^*flox/+*^ and *ROSA*^*mTmG/+*^ mice have been previously described^28, 48, 65, 66^. *Sox2*^*CreERT2/+*^; *Ctnnb1*^*lox(ex3)/+*^; *ROSA26*^*flox-stop-YFP/+*^ and *Sox2*^*CreERT2/+*^; *ROSA26*^*flox-stop-YFP/+*^ mice were induced with a single intra-peritoneal injection of tamoxifen (Sigma, T5648) at a dose of 0.15 mg per gram of body weight. All mouse procedures were performed under licence, following UK Home Office Animals (Scientific Procedures) Act 1986 and local institutional guidelines (UCL ethical review committee). A humane end-point was chosen to avoid severe adverse effects and minimise suffering of mice harbouring tumours, according to UK Home Office and local Ethical Committee regulations. Samples of human ACP were obtained from the Department of Histopathology at Great Ormond Street Hospital for Children and the CCLG tissue bank with full consent (Research Ethics Committee application, REC 14 LO 2265).

### Flow-activated cell sorting

Anterior pituitaries from age- and sex-matched mice were dissociated mechanically into single-cell suspensions following incubation in enzyme mix (0.5% w/v Collagenase type 2 (Lorne Laboratories Ltd.), 0.1× Trypsin (Gibco) and 50 μg/ml DNaseI (Worthington) with 2.5 μg/ml Fungizone (Gibco) in Hank’s Balanced Salt Solution (HBSS (Gibco) for 4 h at 37 °C. Cells were then washed in HBSS and suspended in PBS supplemented with 1% Foetal Calf Serum (PAA), 25 mM HEPES and 1 ng/μl of propidium iodide (PI, BD Pharmigen). Cells were flow sorted for YFP and PI fluorescence using a MoFlo XPD (Beckman Coulter, Fullerton, CA, USA) Flow Cytometer. Fluorescence was detected using a 530/540 filter, where PI positive cells were excluded and the YFP positive and negative fractions collected directly into RLT buffer (Qiagen) supplemented with 1% 2-mercaptoethanol (Sigma) for subsequent RNA isolation.

### Expression analyses in histological sections

Whole-mount X-gal staining, immunostaining and in situ hybridisation on 4–6 µm paraffin sections were performed as previously described^27^. At least three pituitaries or tumours and between two to six non-consecutive slides were analysed. As negative controls, sections were hybridised with sense riboprobes for in situ hybridisation or secondary antibody alone for immunohistochemistry/immunofluorescence. A list of the antibodies and the antigen retrieval methods used is presented in Supplementary Data 1.

### EdU labelling

Pregnant females were injected intra-peritoneally with 100 mg/kg of EdU and embryos dissected 90 min later. After histological processing and sectioning (see above), double immunostaining for β-catenin and EdU was conducted using the Click-It EdU imaging kit (Invitrogen) according to the manufacturer’s instructions.

### RNA extraction and cDNA generation

Flow sorted cells from *Sox2*^*CreERT2/+*^;*Ctnnb1*^*lox(ex3)/+*^;*ROSA26*^*YFP/+*^ and *Sox2*^*CreERT2/+*^;*ROSA26*^*YFP/+*^ mice were processed for total RNA extraction using the RNeasy Micro kit (Qiagen). In total, 5 μl of eluted RNA was then amplified using the Ovation Pico WTA V2 kit (NuGEN), followed by purification of the resultant cDNA using the QIAquick PCR purification kit (Qiagen). Pituitaries from *Hesx1*^*Cre/+*^*;Ctnnb1*^*lox(ex3)/+*^ and control littermates were dissected at 18.5 dpc. The posterior lobe of the pituitary was discarded and the intermediate and anterior lobes were processed for total RNA extraction using the RNeasy Micro kit (Qiagen). Approximately 1 μg of total RNA was reverse transcribed to cDNA using the Transcriptor First Strand cDNA Synthesis Kit and random hexamers (Roche).

### Quantitative real-time PCR and RNA-seq

Quantitative real-time PCR reactions were run in triplicate using the iTaq SYBR Green (BIORAD) and replicated using a minimum of four independent samples for each genotype. Primer sequences are included in Supplementary Data 5. For *Shh*, we used the Qiagen QuantiTect Primer Assay (Cat. no. 249900). Results were analysed using the ΔΔCt method. For RNA-Seq of *Sox2*^*CreERT2/+*^;*Ctnnb1*^*lox(ex3)/+*^;*ROSA26*^*YFP/+*^ and *Sox2*^*CreERT2/+*^;*ROSA26*^*YFP/+*^ flow-sorted YFP+ cells, isolated RNA was processed by the SMARTer V4 low input assay kit (Clontech) to generate amplified cDNA using the strand-switching protocol. Library preparation was performed with 200 pg of amplified cDNA using the Nextera XT preparation kit with 12 cycles of PCR. Sequencing was then performed on an Illumina NextSeq 500 with 43 bp paired end reads. Sequencing data was then aligned in BaseSpace. FASTQ and BAM files are available on Array Express (E-MTAB-5538), Differential expression was performed using DESeq2^67^. For GSEA, genes were ranked by the Wald statistic and GSEA performed using the pre-ranked tool in GSEA v2.2 (Broad Institute)^68^. The WNT pathway gene set was downloaded from the hallmark molecular signatures database v5.2 (Broad Institute)^68^.

### Analysis of the relationship between murine and human ACP

To explore the relationship between the ACP models and human ACP, gene sets of the 100 and 500 most upregulated genes in human ACP were generated and GSEA performed as above. In addition, expression data (normalised counts or intensity values) of 11,347 genes, for which expression data was available between non-cluster and cluster cells from human ACP (array express accession no: E-MTAB-5266), *Sox2*^*CreERT2/+*^*;Ctnnb1*^*lox(ex3)*^*;R26*^*YFP/+*^ tamoxifen-induced mice (E-MTAB-5538) and *Hesx1*^*Cre/+*^*;Ctnnb1*^*lox(ex3)/+*^ (E-MEXP3492) animals were median scaled and merged. Clustering was performed using Spearman’s correlation and Ward’s algorithm.

### Exome sequencing of mouse tumours

Fully developed pituitary tumours and matched tail samples of 15 *Hesx1*^*Cre/+*^*;Ctnnb1*^*lox(ex3)/+*^ mice were collected at humane end-points. DNA was extracted using the DNeasy bloods and tissue kit (Qiagen) and exonic DNA was captured using the Agilent whole exome capture kit (SureSelect Mouse All Exon). Captured material was indexed and sequenced on the Illumina platform at the Wellcome Trust Sanger Institute at 30× depth. Raw pair end sequencing reads were aligned with BWA-mem to the GRCm38 mouse reference genome. Duplicated reads were marked using biobambam. Somatic variants were detected using CaVEMan, an expectation maximisation-based somatic substitution-detection algorithm. Detected somatic variants were then filtered using an array of quality filters and common mouse genome variants were excluded^69, 70^. A sub-selection of 14 genes, where mutant variant allele frequencies were greater than 20% and at least three mutant reads were detected, were validated by Sanger sequencing. Primer were designed in flanking regions (Supplementary Data 2). Sequencing of the PCR fragments was performed by Source BioScience (UK).

### Semi-quantitative ELISA cytokine arrays

Mouse C1000 cytokine arrays (AAM-CYT-1000-4, RayBiotech) were used to quantify the expression of SASP factors in protein extracts from whole pituitaries dissected at 18.5 dpc according to manufacturer’s instructions (sex was not determined in the embryos). For each experimental sample, total protein was extracted from four pituitaries using the cell lysis solution provided in combination with a TissueRuptor (Qiagen) and passed through a Qiashredder column (Qiagen). Total protein was then measured using BradfordUltra reagent (Expedeon). ELISA membranes were blocked for 30 min using blocking buffer provided by the manufacturer and then incubated overnight at 40 °C with 70 μg of total protein. Following extensive washes, membranes were incubated with biotinylated antibodies and developed according to manufacturer’s indications. Chemiluminescence detection was conducted with a ChemiDoc gel documentation system (BioRad) and imaged using ImageLab Software (Version 5.2.1, BioRad). Analysis of high-resolution images from each membrane was conducted using Fiji/ImageJ and the Protein Microarray Analysis macro (IRB, Barcelona). Calculation of fold changes in expression was conducted according to manufacturer’s instructions.

### Assessment of clonogenic potential

Anterior pituitaries were dissected and enzyme treated as described for flow-activated cell sorting, above. This was followed by mechanically dissociation into a single-cell suspension and subsequent washing in Hank’s Balanced Salt Solution. Cells were placed directly into Adherent Pituitary Stem Cell medium (DMEM-F12; Gibco) containing 5% Foetal Calf Serum (PAA), 20 ng/ml bFGF (R&D Systems) and 50 ng/ml cholera toxin (Sigma). Cells were plated into six-well plates at a density of 1000, 2000 and 4000 cells per well for drug or vehicle treated. After seven days in culture, adherent colonies were fixed, washed with PBS and stained using Meyer’s Haematoxylin (Sigma) for 10 min and counted manually.

### Ex vivo culture of mouse pituitaries and ABT-737 treatment

For ex vivo pituitary culture, anterior pituitaries were isolated from *Hesx1*^*Cre/+*^*;Ctnnb1*^*lox(ex3)/+*^ embryos at 18.5 dpc, placed on top of 0.2 µM Whatman filters (SLS) in 24 well plates containing 500 µl of media (DMEM-F12 (Gibco), 1% Pen/Strep (Sigma) and 1% FBS (PAA) supplemented with either ABT-737 at 2.5 µM (Adooq Bioscience), ABT-263 at 1 µM (Caymen Chemical) or vehicle. Anterior pituitaries were cultured for 3–4 days, changing media every day and subsequently processed for histological analysis.

### Statistical analyses

Results are presented as means ± s.e.m. unless otherwise indicated. One-tailed Student’s *t* test was performed to compare the means of two groups. Significance was assumed at *P* < 0.05. Sample size varied, but a minimum of three biological replicas were used for analysis. Statistical analyses were performed using GraphPad software. No samples were excluded from analyses. No formal blinding or randomisation was used.

### Code availability

Codes are available upon request

## Electronic supplementary material

Supplementary Information

Description of Additional Supplementary Files

Supplementary Data 1

Supplementary Data 2

Supplementary Data 3

Supplementary Data 4

Supplementary Data 5
