# Supplementary material for: Stem cell senescence drives age-attenuated induction of pituitary tumours in mouse models of paediatric craniopharyngioma
Source: Nat Commun. 2017 Nov 28;8:1819. doi: 10.1038/s41467-017-01992-5 (PMC5703905; doi:10.1038/s41467-017-01992-5)
Supplement: Supplementary file 1 — Supplementary Information [file 41467_2017_1992_MOESM1_ESM.pdf]

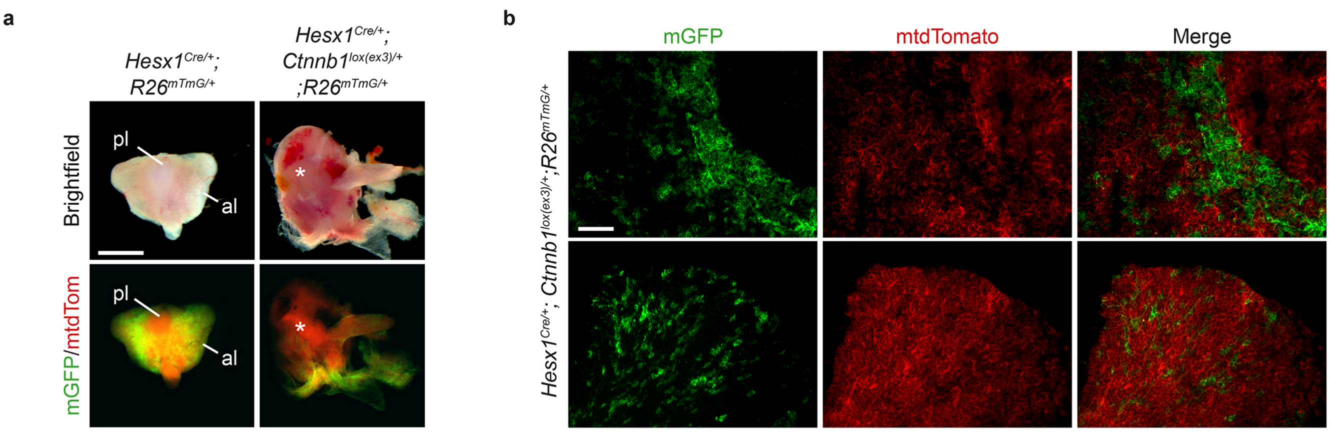

Supplementary Figure 1. Genetic tracing of *Hesx1$^{Cre/+}$;Ctnnb1$^{lox(ex3)/+}$;R26$^{mTmG/+}$* tumours. (**a**) Representative brightfield and epifluorescence pictures of a *Hesx1$^{Cre/+}$;R26$^{mTmG/+}$* control pituitary and a *Hesx1$^{Cre/+}$;Ctnnb1$^{lox(ex3)/+}$;R26$^{mTmG/+}$* pituitary tumour. Note that in the control, the anterior lobe (al) is mostly GFP+ve and the posterior lobe (pl) is tdTomato+ve. In the tumours, most of the cells express tdTomato whereas GFP-expressing cells are restricted to a small region. Scale bar: 1 mm. (**b**) Histological analysis of cryo-sections of two *Hesx1$^{Cre/+}$;Ctnnb1$^{lox(ex3)/+}$;R26$^{mTmG/+}$* tumours showing that most of the tumour cells express tdTomato, but sporadic areas of cells expressing GFP can be observed. Scale bars: 100 μm.

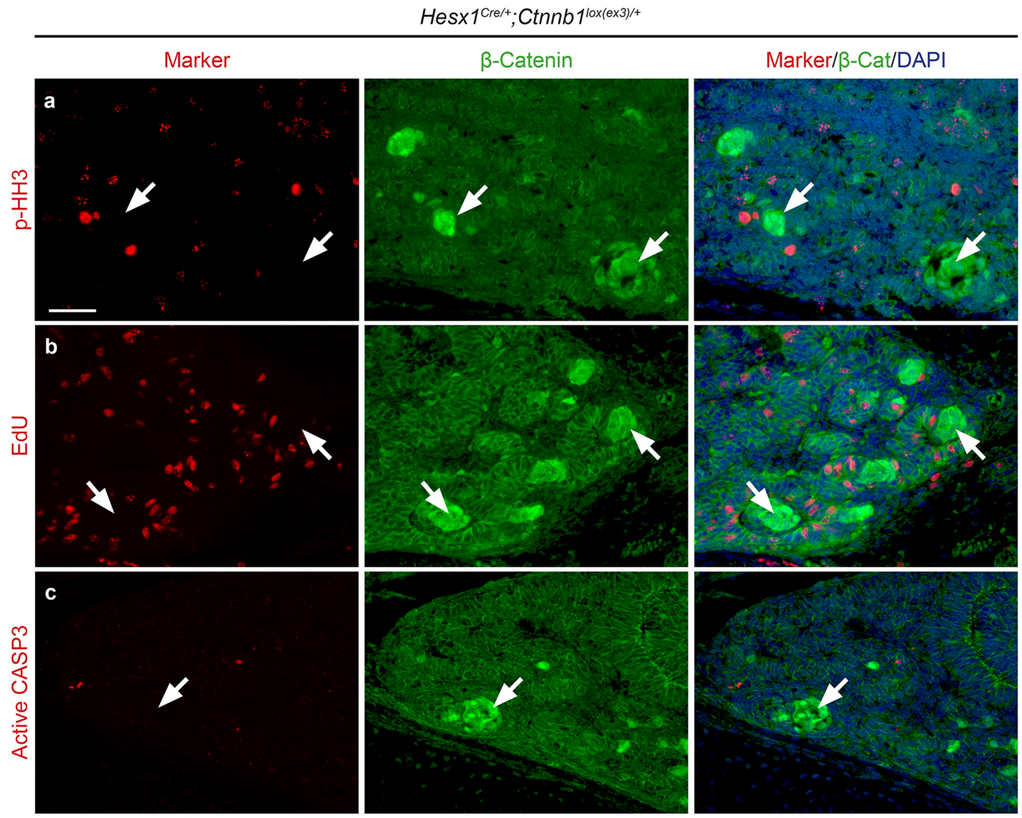

Supplementary Figure 2. β-catenin-accumulating clusters in the *Hesx1*$^{Cre/+}$*;Ctnnb1*$^{lox(ex3)/+}$ mice are non-proliferative and viable. Immunostaining against phopho-histone H3 and active caspase3 showing the absence of mitosis and apoptosis in the cluster cells (arrows) at 18.5 dpc. The nucleotide analogue EdU is also excluded from cluster cells, suggesting that these cells are not replicating their DNA. Scale bar: 50 μm.

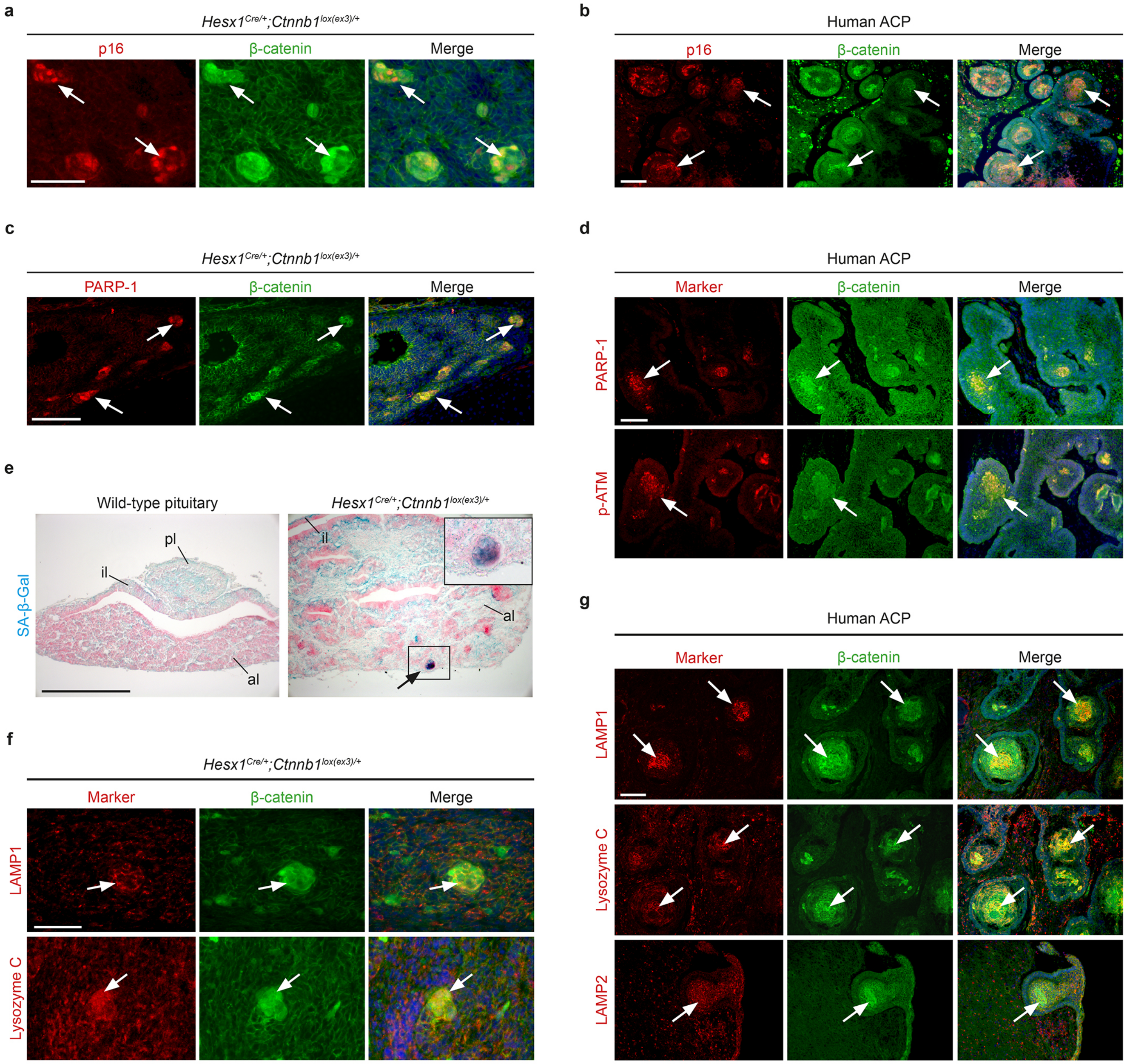

Supplementary Figure 3. Cluster cells in *Hesx1*$^{Cre/+}$*;Ctnnb1*$^{lox(ex3)/+}$ pre-tumoural pituitaries and human ACP express senescent markers. (**a**,**b**) Double immunostaining revealing the expression of the cell cycle inhibitor p16 in cluster cells (arrows). (**c**,**d**) Expression of PARP1 and phospho-ATM demonstrating the activation of the DNA-damage response in cluster cells (arrows). (**e**) Senescence-associated β-Gal staining showing staining in small group of cells resembling the β-catenin-accumulating cell clusters (arrow). The inset depicts a higher magnification of the boxed area. 'pl', 'il' and 'al' are posterior, intermediate and anterior lobes, respectively. (**f**,**g**) Double immunostaining revealing the expression of lysosomal markers in the clusters (arrows) in both mouse pre-tumoural pituitaries and human ACP. Scale bars: a,d,f: 50 µm; b,c,g: 100 µm; e: 500 µm.

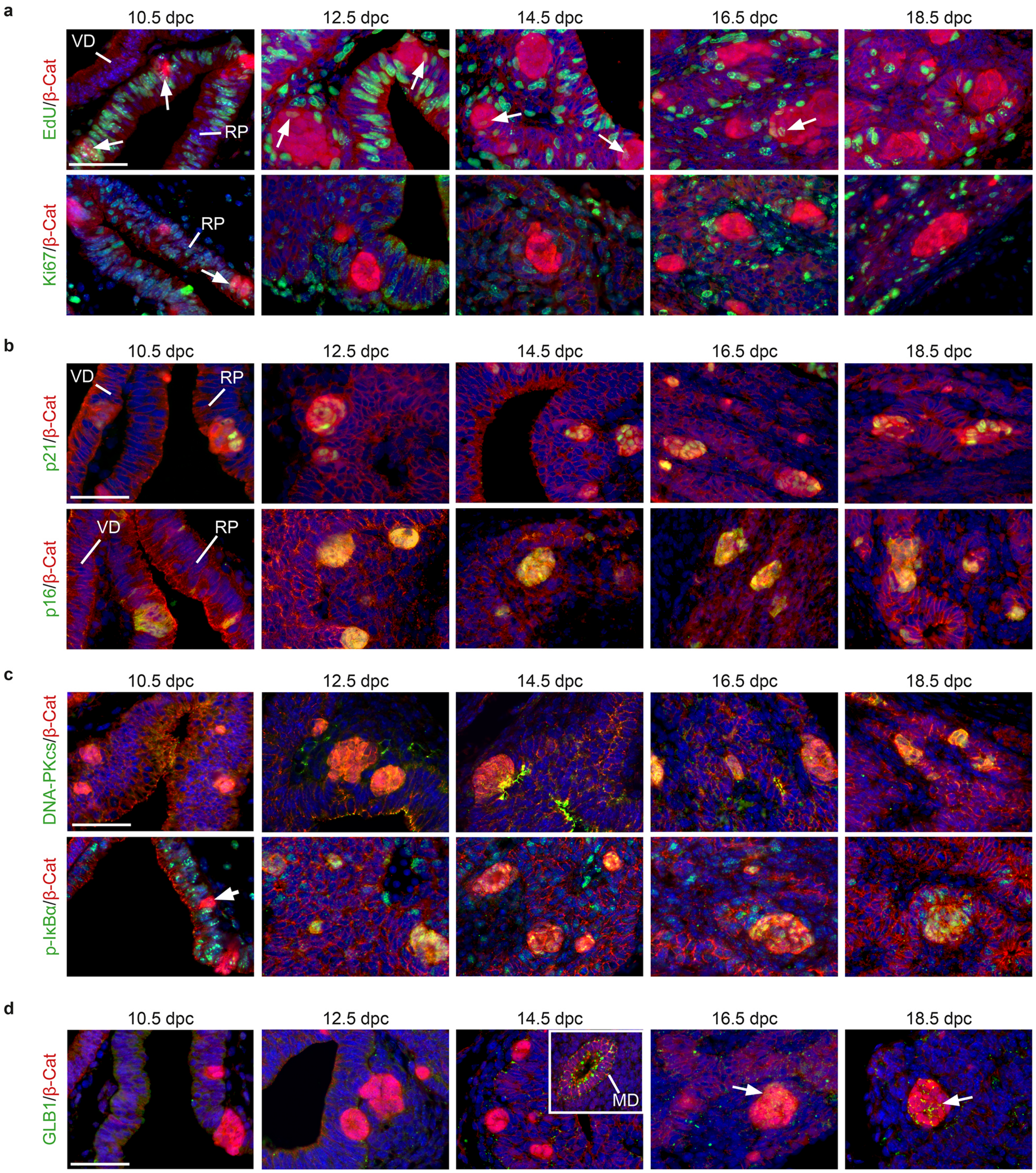

Supplementary Figure 4. Expression of senescence markers and β-catenin in the *Hesx1*$^{Cre/+}$*;Ctnnb1*$^{lox(ex3)/+}$ developing pituitaries from 10.5 to 18.5 dpc. (**a**) Double immunostaining showing incorporation of EdU and Ki67 expression in cluster cells. Note that Ki67 immunoreactivity and EdU staining is more abundant at early stages of development (arrows). A low level of EdU incorporation is seen up to 16.5 dpc in sporadic cluster cells, perhaps due to the DNA repair machinery or endoreplication. (**b**) The expression of the cell cycle inhibitor p21 in cluster cells increases as development progresses, whilst p16 is abundantly expressed at all stages analysed. Scale bar: 50 µm. (**c**) Expression of phospho-DNA-PKcs and phosphor-IkBa is detected in the cluster cells from early stages. 50 µm. (**d**) Immunostaining against the lysosomal marker GLB1 (encoding senescence-associated β-galactosidase) showing strong punctate signal only at 16.5

and 18.5 dpc (arrows). The inset depicts GLB1 expression in the mesonephric tubules of the same embryo. Scale bar: 50 μm.

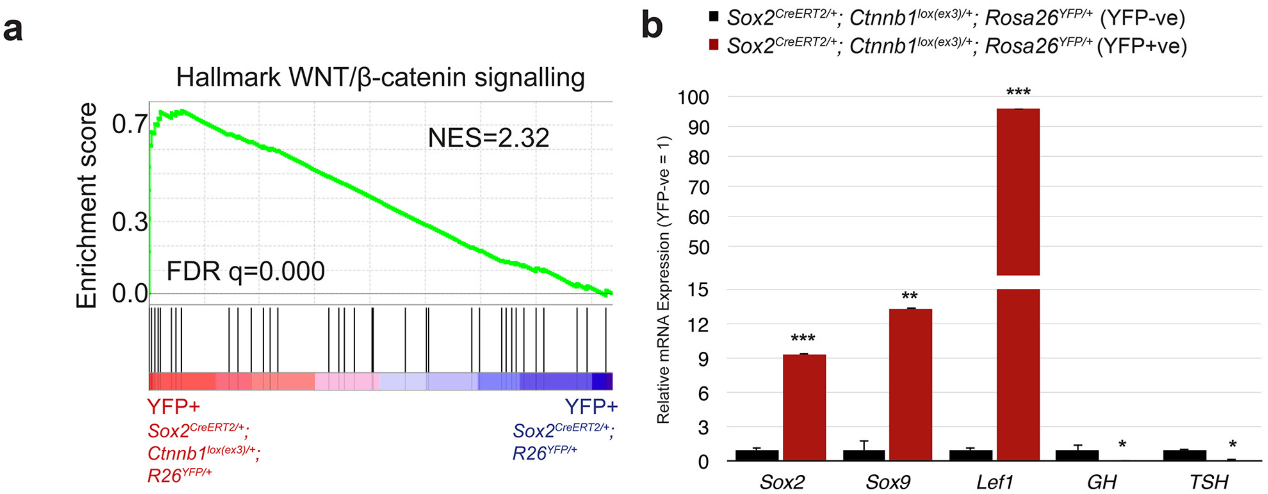

Supplementary Figure 5. **β-catenin-accumulating cluster cells are contained within the YFP+ve cell population in** ***Sox2**$^{CreERT2e/+}$**;Ctnnb1**$^{lox(ex3)/+}$**;R26**$^{YFP/+}$* **pituitaries.** (**a**) Gene set enrichment analysis (GSEA) of YFP+ve cells isolated by fluorescence-activated cell sorting (FACS) from either from *Sox2*$^{CreERT2e/+}$*;Ctnnb1*$^{lox(ex3)/+}$*;R26*$^{YFP/+}$ mutant or *Sox2*$^{CreERT2e/+}$*;R26*$^{YFP/+}$ control pituitaries. There is a significant enrichment of the molecular profile of the mutant relative to the control YFP+ve cells against a data set of activated WNT/β-catenin signalling, suggesting that the β-catenin cluster cells are contained in the mutant YFP+ve population. (**b**) qRT-PCR showing up-regulation of the pituitary stem cells factors *Sox2* and *Sox9* and the WNT target *Lef1,* as well as down-regulation of markers of terminally-differentiated cells (*Gh*, growth hormone and *Tsh*, thyroid-stimulating hormone) in YFP+ve relative to YFP-ve cells isolated by fluorescence-activated cell sorting of *Sox2*$^{CreERT2e/+}$*;Ctnnb1*$^{lox(ex3)/+}$*;R26*$^{YFP/+}$ pituitaries induced with tamoxifen at 4 weeks of age. Bars represent the mean, and error bars represent standard error of the mean (SEM) of 3 biological replicates., * p<0.05, ** p<0.01, ** p<0.001.

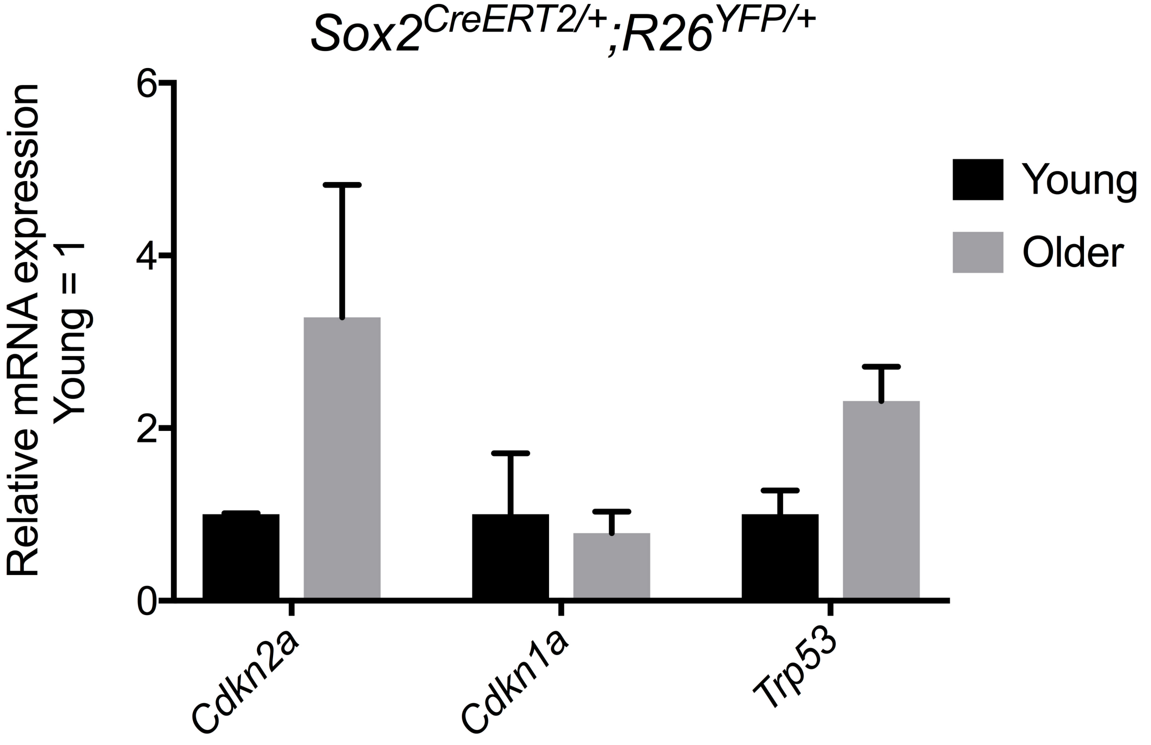

Supplementary Figure 6. Sox2+ve cells from younger mice express lower levels of senescence markers. *Sox2*$^{CreERT2/+}$*;R26*$^{YFP/+}$ mice were tamoxifen induced at either 1 or 6 months of age (young and older mice, respectively). The anterior pituitary was collected 4 weeks post-induction and processed for molecular analysis of YFP+ve cells isolated by fluorescence-activated cell sorting (FACS). qRT-PCR showing lower levels of expression of the senescence markers *Cdkn2a* (*p16*) and *Trp53* (*p53*) in the younger relative to the older YFP+ve cells of *Sox2*$^{CreERT2e/+}$*;R26*$^{YFP/+}$ pituitaries. Bars represent the mean, and error bars represent standard error of the mean (SEM) of 5 biological replicates from the young cohort and 4 biological replicates from the older cohort.

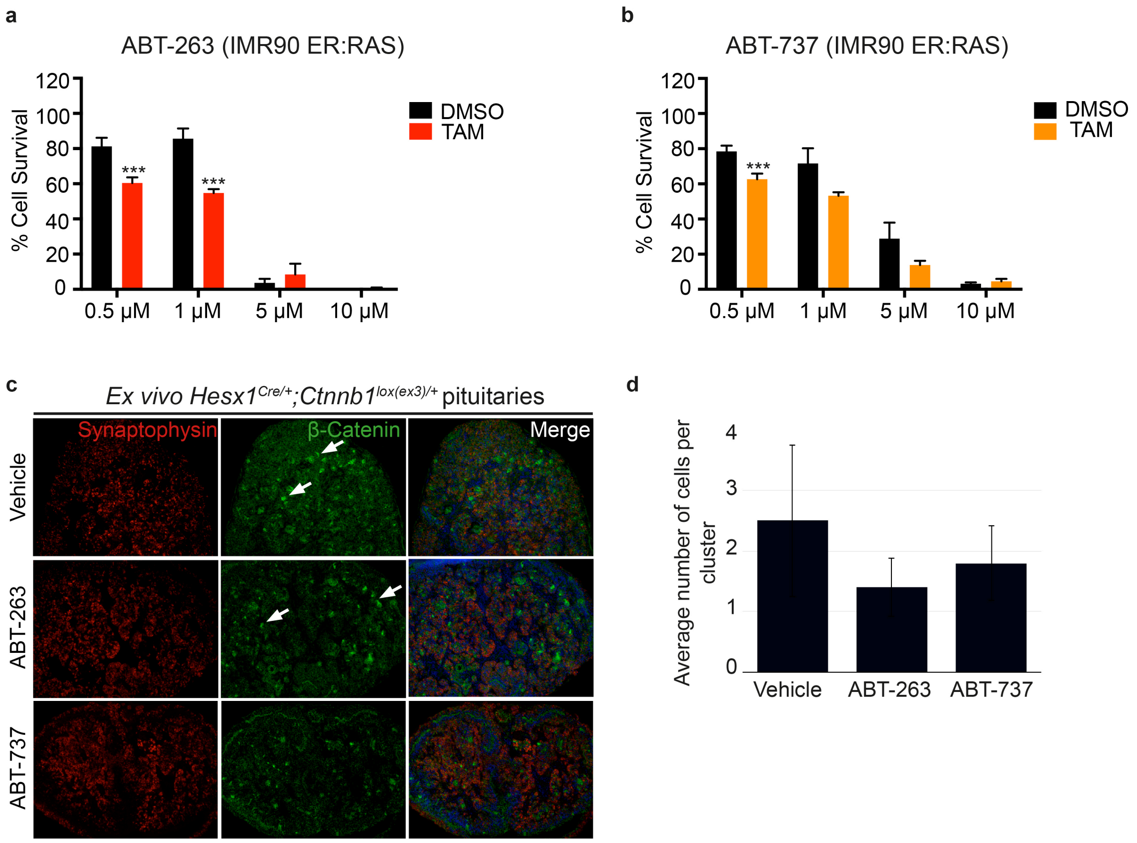

Supplementary Figure 7. *In vitro* testing of the senolytics ABT-263 and ABT-737. (**a,b**) IMR90 ER:RAS cells were cultured in the presence of either tamoxifen (TAM), to induce the expression of oncogenic KRAS leading to senescence induction, or DMSO (control non-senescent cells). The effect of the senolytics ABT-263 and ABT-737 was assessed by culturing induced (red/orange bars) and un-induced (black bars) cells in media containing increasing concentrations of these compounds (0.5-10 mM). For ABT-263, only 0.5 mM and 1 mM resulted in a specific decrease in numbers of senescent cells (red/orange bars) compared with the non-senescent (black bars) controls (0.5mM, 25.7%; 1mM, 35.9%), suggesting specific ablation of the former. For ABT-737, 0.5 mM and 1 mM caused a reduction, that reached significance only for 0.5 mM (0.5mM, 20.1%; 1mM, 25.7%). At 5 mM and 10 mM numbers of senescent and non-senescent cells were reduced, suggesting toxicity. (**c, d**) Pituitaries from *Hesx1$^{Cre/+}$;Ctnnb1$^{lox(ex3)/+}$* mice at 18.5 dpc were cultured *ex-vivo* in the presence of 1 mM of the ABT senolytic compounds for 4 days and analysed histologically. Specific immunostaining against synaptophysin and β-catenin revealing the presence of the non-cluster and cluster cells, respectively. Note that quantitative analysis showed a reduction in the average number of cells per cluster, which did not reach significance (number of cells/cluster: (vehicle 2.46±1.26, 41 clusters counted from 3 pituitaries; ABT-263, 1.4±0.49, p=0.47, 40 clusters counted from 4 pituitaries; ABT-737, 1.8±0.62, p=0.70, 38 clusters counted from 3 pituitaries; Student's t-test). Bars in a, b and d represent the mean, and error bars represent standard error of the mean (SEM) of 3 biological replicates. *** p<0.001, Student's t-test.

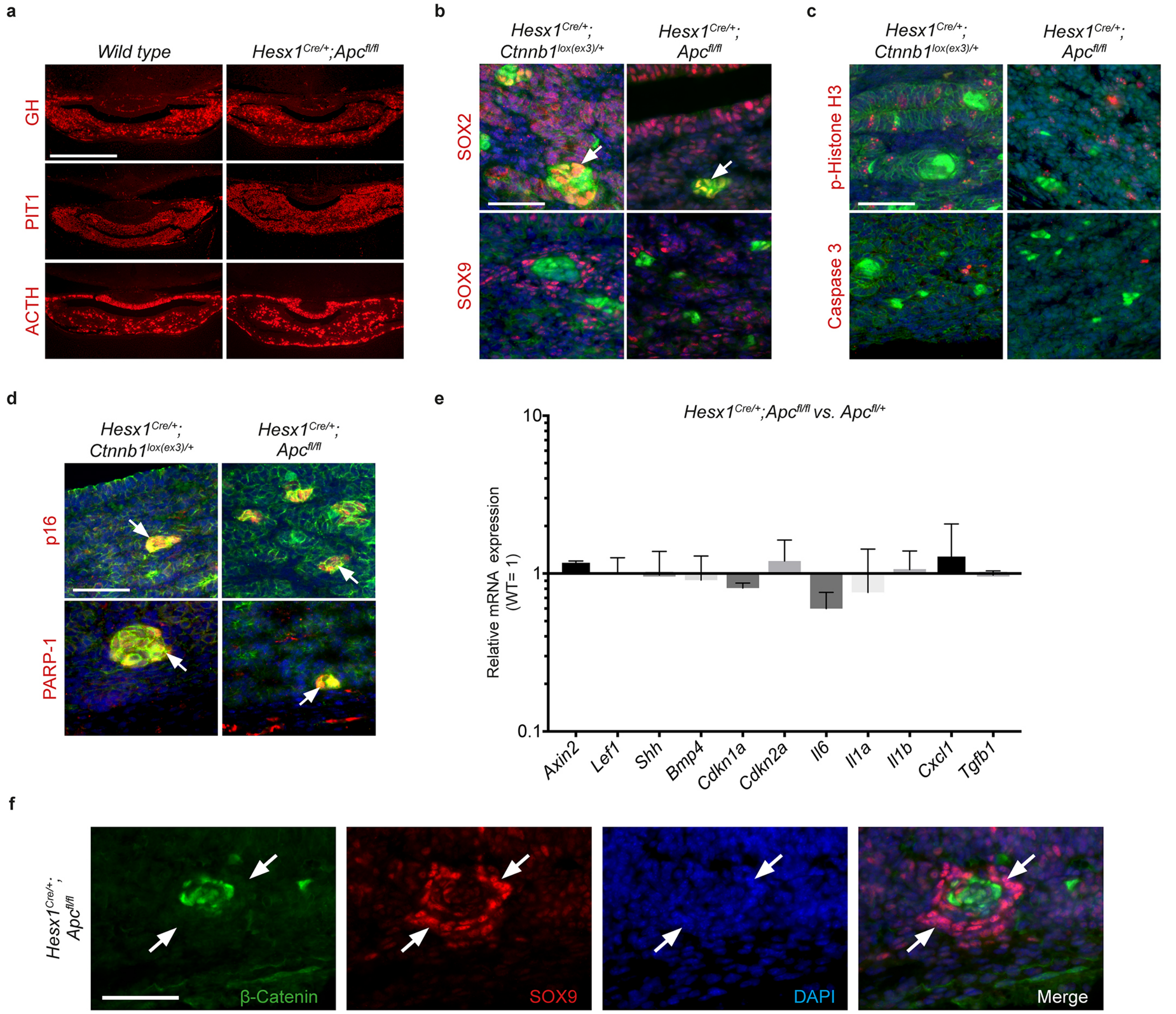

Supplementary Figure 8. Analysis of *Hesx1*$^{Cre/+}$;*Apc*$^{fl/fl}$ pituitaries. (**a**) Immunostaining against the pituitary markers growth hormone (GH), the cell-lineage marker PIT1 and adrenocorticotropic hormone (ACTH) revealing comparable expression in the *Hesx1*$^{Cre/+}$;*Apc*$^{fl/fl}$ pituitaries and wild-type control pituitaries. (**b**-**d**) Double immunostaining demonstrating that cluster cells express SOX2, p16 and PARP1 (arrows), but not SOX9, phospho-histone H3 and active Caspase 3. (**e**) qRT-PCR analysis showing non-significant differences in the expression of senescence and SASP factors between *Hesx1*$^{Cre/+}$;*Apc*$^{fl/fl}$ and *Apc*$^{fl/+}$ pituitaries. Bars represent the mean, and error bars represent standard error of the mean (SEM) of 3 biological replicates. Student's t-test. (**f**) Accumulation of SOX9+ve cells around the clusters is observed only when *Hesx1*$^{Cre/+}$;*Apc*$^{fl/fl}$ clusters reach a similar size to those in the *Hesx1*$^{Cre/+}$;*Ctnnb1*$^{lox(ex3)/+}$ pre-tumoural pituitaries. Scale bars: a: 500 μm; b,c,d,f: 50 μm.

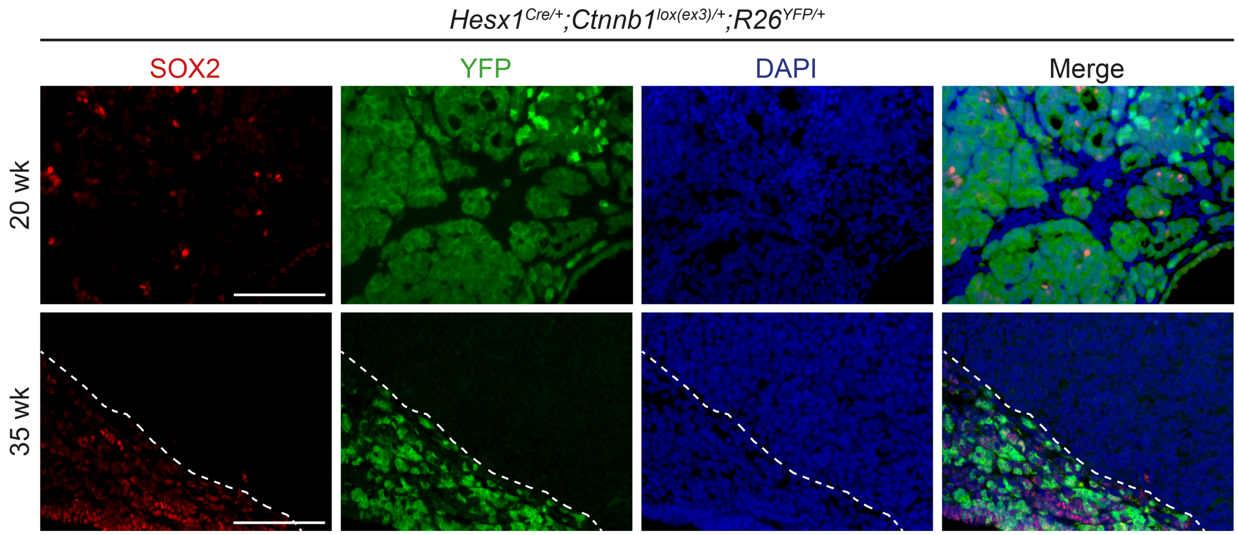

Supplementary Figure 9. Expression analysis of SOX2 in *Hesx1*$^{Cre/+}$*;Ctnnb1*$^{lox(ex3)/+}$*;R26*$^{YFP/+}$ postnatal pituitaries and tumours. Double immunostaining for SOX2 and YFP in 20 week-old pre-tumoural pituitaries showing that SOX2 is only expressed in the YFP+ve compartment. In 35 weeks-old fully-developed tumours (lower row), SOX2 is not expressed in the growing YFP-ve regions, while it is expressed in the displaced regions that contain YFP+ve cells. Results are representative of 3 different pituitaries/tumours from each stage. Scale bars: 100 µm.
