# Supplementary material for: Stem cell senescence drives age-attenuated induction of pituitary tumours in mouse models of paediatric craniopharyngioma
Source: Nat Commun. 2017 Nov 28;8:1819. doi: 10.1038/s41467-017-01992-5 (PMC5703905; doi:10.1038/s41467-017-01992-5)
Supplement: Supplementary file 2 — Description of Additional Supplementary Files [file 41467_2017_1992_MOESM2_ESM.pdf]

Description of Additional Supplementary Files

File Name: Supplementary Data 1
Description: List of antibodies used in this study.

File Name: Supplementary Data 2
Description: Results of exome sequencing of mouse ACP tumours.

File Name: Supplementary Data 3
Description: RNA-Seq data from FACS isolated YFP+ve cells from *Sox2*$^{CreERT2/+}$*;Ctnnb1*$^{lox(ex3)/+}$*;R26*$^{YFP/+}$ mutant pituitaries and *Sox2*$^{CreERT2/+}$*;R26*$^{YFP/+}$ controls.

File Name: Supplementary Data 4
Description: Mendelian ratios from *Hesx1*$^{Cre/+;}$*Apc*$^{fl/fl}$ x *Apc*$^{fl/+}$ genetic crosses.

File Name: Supplementary Data 5
Description: List of primers used for qRT-PCR.
